# Real-world outcomes from 2,905 episodes of hospital at home care: a propensity-matched cohort study

**DOI:** 10.3389/fdgth.2026.1716319

**Published:** 2026-04-08

**Authors:** Michael Shaw, Batool Almogheer, Dominique Auger, Andrew Barlow, Balasmita Bhaskaran, Maria Buxton, Marco Cerulli, Kalpana Giri Ghimire, Edward Hiller, Zoe Jayne, Michal Kelly, Matthew Knight, Eleanor Zinkin, Niall G. Keenan

**Affiliations:** 1 West Hertfordshire Teaching Hospitals NHS Trust, Watford, United Kingdom; 2 Imperial College Healthcare NHS Trust, London, United Kingdom; 3 Central London Community Healthcare NHS Trust, London, United Kingdom; 4 East Suffolk and North Essex NHS Foundation Trust, Colchester, United Kingdom; 5 Imperial College, London, United Kingdom

**Keywords:** cost-effectiveness, hospital at home, length of stay, virtual hospital, virtual ward

## Abstract

**Background:**

Hospital at home (HAH) services within the UK have expanded rapidly over the last 5 years, but there is comparatively little evidence demonstrating their clinical effectiveness. In this study, we evaluated the clinical outcomes, safety, and cost-effectiveness of a comprehensive HAH service in England.

**Methods:**

We conducted a retrospective cohort study of patients admitted to our HAH service between December 2021 and May 2024, including pathways for heart function, airway disease, and acute respiratory infection. A 1:1 propensity score matched control cohort of patients admitted to inpatient care was created, using regression adjustment to derive doubly robust estimates of main outcomes. Primary outcomes included length of stay and total bed-day costs. Secondary outcomes included 30-day readmission rates, 90-day mortality, and patient experience and acceptability metrics.

**Results:**

We analysed 2,972 HAH episodes, yielding, after exclusions, a total of 1,488 inpatient-originated (IP) episodes that were matched 1:1 to controls, as well as 754 admission prevention episodes for a separate analysis. HAH reduced length of stay compared with matched inpatient controls (bed-day savings: 3.13 days, 95% CI 2.60–3.67, *p* < 1 × 10^−29^). Total bed-day savings were 13,119 days, yielding net savings of £3.79 million over 33 months.

All-cause 30-day readmission rates were significantly lower in HAH cohorts than in matched controls (OR 0.55, 95% CI 0.42–0.70, *p* < 3 × 10^−6^), as was total time in hospital over 90 days from initial presentation (2.64 days fewer, 95% CI 1.87–3.40, *p* < 2 × 10^−11^) and 90-day all-cause mortality (OR 0.43, 95% CI 0.35–0.53, *p* < 3 × 10^−16^).

**Conclusions:**

This large real-world evaluation demonstrates that HAH services significantly reduce length of stay, readmissions, and healthcare costs while maintaining safety and possibly reducing mortality. These findings support a wider implementation of HAH.

## Introduction

Hospital at home (HAH) models of care are well established globally (1, 2); in fact, this model of care predates the concept of the hospital itself (3). The COVID-19 pandemic led to a significant acceleration of this model of care, driving an expansion of hospital at home care within the UK, initially driven by a need to rapidly expand capacity and address concerns that the traditional inpatient model—sometimes referred to as “brick-and-mortar’ (BAM)—would be overwhelmed with patients with COVID-19 infection. Experience has shown that these models of care are safe, clinically effective, cost-effective, and highly acceptable to patients. The Darzi Report, a recent critical review of the NHS in England, has lauded the innovation of the virtual ward programme, and the Secretary of State for Health and Social Care has identified three priorities for the NHS in England (analogue to digital, hospital to community, and treatment to prevention), the first two of which clearly align with the Virtual Ward agenda (4). Furthermore, the NHSE 10 Year Plan published in July 2025 makes this even more explicit (5). However, there are some negative reports in the literature, with some studies failing to show a clinical impact or suggesting greater costs of care in a hospital at home model (6, 7). The contentious nature of the debate has led some healthcare leaders to question the entire Virtual Ward programme (8).

The West Hertfordshire Teaching Hospitals NHS Trust (WHTHT) established the first HAH service for COVID-19 in the UK early in 2020 and cared for over 7,000 patients infected with COVID-19 using remote monitoring. In December 2021, the South and West Herts Health and Care Partnership (SWH)—a partnership that includes the WHTHT and our local community healthcare provider, Central London Community Healthcare NHS Trust (CLCH)—launched an HAH service for airway disease (ABC: asthma, bronchiectasis, COPD), heart failure (HF), and acute respiratory infection (ARI). Each pathway accepted admission prevention (AP) referrals from community providers, as well as accepting referrals from, and actively seeking, suitable BAM hospital inpatients. BAM inpatients were accepted both in their early stage (admission diversion—AD) and in their care journey (early supported discharge—ESD).

After 33 months of operation, we performed an analysis of our virtual ward, focusing on clinical outcomes, safety, and cost-effectiveness. This is the largest published analysis of a hospital at home programme in the UK to date and one of the largest globally.

## Methods

### Hospital at home care

The HAH service is a partnership between WHTHT acute health services and CLCH community care. It combines hospital- and community-based doctors, nurses, pharmacists, and allied health professionals, with administrative support. Remote monitoring devices such as the Masimo Radius PPG tetherless pulse oximeter (Masimo, Irvine, CA, USA) are linked to a centralised “real-time” monitoring hub situated in the acute hospital. Reviews are performed remotely or face to face in patients' homes as indicated. The service functions 7 days a week and aims to replicate key aspects of inpatient care, including real-time monitoring of patient's observations, daily home visits where required, and rapid access to imaging and pathology services. The HAH service aims to optimise utilisation of the inpatient bed base and provide safe and timely care in the patient's home.

### Patient selection

Inpatient selection is driven by referrals from the emergency department (ED), acute medical receiving unit, and all other inpatient ward areas; by clinically led screening of patients identified by automated electronic patient record (EPR) queries (including use of prescriptions for nebulisers, intravenous furosemide, and antibiotics for respiratory infections); and by proactive screening of patients in the ED and acute medical receiving wards.

Community patient selection is done through referrals from respiratory and cardiac community specialist teams, general practitioners, paramedics, and occasionally from the NHS 111 hotline service.

### Hospital at home inclusion and exclusion criteria

Inclusion criteria are pathway dependent as outlined below (Table 1). Exclusion criteria across all pathways include inability to deliver required medical and care needs at home, patient refusal, inability to manage self-monitoring, and physical or mental difficulties impairing the ability to seek medical attention in case of clinical deterioration.

**Table 1:** Inclusion and exclusion criteria.

Pathway	Inclusion Criteria	Exclusion Criteria
HF	Diagnosis of Heart Failure requiring fluid status optimisation	Diagnosis of Severe Aortic Stenosis
	Diagnosis of Atrial Fibrillation, or atrial flutter, requiring rate or rhythm control optimisation	
ABC	Diagnosis of Asthma, Bronchiectasis, or COPD	Diagnosis of ILD (initially)
ARI	Diagnosis of Pneumonia, Viral Pneumonitis, or COVID-19	No specific criteria
	Diagnosis of other medical problems deemed suitable for remote management	
Overall	Pathway Specific Criteria	Inability to deliver required medical and care needs at home
		Patient refusal
		Inability to manage self-monitoring
		Physical or mental difficulties impairing the ability to seek medical attention in case of deterioration

Pathway-specific and whole-service inclusion and exclusion criteria during the study period.

### Heart failure pathway

Patients suffering from heart failure requiring fluid status optimisation and medical optimisation of guideline-directed therapy and those experiencing atrial fibrillation or atrial flutter requiring optimisation of rate or rhythm control were considered for the Heart Failure pathway. Patients with severe aortic stenosis (AS) were initially excluded from the pathway; however, over time, patients with severe AS awaiting urgent intervention were considered for the HAH pathway after careful selection by the transcatheter aortic valve implantation (TAVI) service consultant.

### Asthma, bronchiectasis, and COPD pathway

Patients with primary diagnoses of asthma, bronchiectasis, COPD, or other chronic respiratory conditions were considered for the ABC pathway. Patients suffering from interstitial lung disease (ILD) were initially excluded because of perceived risk, but from the year 2024, they were included after an agreement with senior clinicians.

### ARI pathway

Patients suffering from pneumonia, lower respiratory tract infections, viral pneumonitis, COVID-19, and other medical conditions deemed suitable for remote management were considered for the ARI pathway. There were no pathway-specific exclusion criteria.

### Onboarding

In the early supported discharge (ESD) and admission diversion (AD) groups, initial assessment, explanation of care model, consent to care, and provision of monitoring equipment were performed by nursing staff at onboarding. Specialist community team reviews were also organised for the first day of HAH care (the day following hospital discharge) for patients with exacerbations of chronic cardiac or respiratory conditions.

AP patients identified in the community were assessed by referring practitioners (including CLCH specialist nurses, physiotherapists, and paramedics) and discussing with the virtual hospital hub; if deemed suitable, these patients were onboarded in the community and initial medical treatment started. The community teams supplied the patients with monitoring equipment. Medical investigations, relevant blood tests, imaging, and a full medical assessment and plan were initiated by the hub medical team for all patients within 24 h of agreement to onboard.

### Patient care

Daily virtual ward rounds for all HAH patients took place with the medical and nursing teams. Each pathway conducted multidisciplinary team (MDT) meetings for soliciting senior and specialist advice at least once a week.

All patients received daily nursing review calls to monitor progress and identify any concerns requiring escalation to the medical team. The frequency of medical review calls was individualised based on patient need and acuity, typically every 2–4 days. The ward round could trigger an at home assessment by a specialist team as indicated.

Outpatient investigations (including bloods and imaging) were available on a same day basis with target timescales matching BAM hospital inpatients.

### Analysis inclusion criteria

All patients admitted under HAH between the service opening on 22 December 2021 and 1 May 2024 were included in the analyses, and all clinical episodes between 1 January 2020 and 15 September 2024 were retrieved. A control group was drawn from a cohort of all inpatients admitted with selected primary or secondary ICD10 diagnoses determined by clinical coding^1^ who had never been admitted to a virtual hospital, and as with HAH patients, all clinical episodes between 1 January 2020 and 15 September 2024 were retrieved. Control patients who had previously been managed under HAH were excluded. This control group was used as a source for controls for propensity-score matching, as described later.

Across both groups, episodes were assessed for data quality, and mislabelled day-case or duplicated episodes were removed and overlapping episodes merged. Episodes were also excluded if an earlier HAH episode existed in the preceding 90 days, the patient was missing necessary data, under the age of 16 at onboarding, discharged after the cut-off date of 1 May 2024 (selected to ensure at least 90 days of follow-up), or died within 24 h of discharge (due to typical variation between recorded time of death and administrative discharge due to mortality).

A consort diagram was generated to illustrate case flow and stepwise exclusions for all care episodes, and a second diagram demonstrated group classification of HAH episodes. For reproducibility, figures for each station on the diagram were derived automatically at each operation modifying the cohort.

The ICD10 inclusion list was derived from an extract tabulating primary ICD10 diagnoses for inpatient source admissions for HAH episodes. This list was reviewed by one author (MS), and diagnoses outside the clinical purview of the ABC, ARI, and HF HAH pathways or with very low incidence (<1%) were removed.

In addition, a set of inpatients who had declined an offer of HAH care for non-medical reasons were included as a quasi-experimental control group. These patients were included following a review of documentation by one author (MS), if contemporaneous records indicated that the patient had been screened and medically accepted for HAH care, there were no clear contraindications to HAH care, and the reason for declining was non-medical. Patient-specified reasons for refusing HAH Care in this cohort have been explored separately (9).

### Data collection

Data on demographics, care episodes, ICD10 clinical coding for each episode, clinical frailty scores, acuity, and observations were obtained for all patients meeting inclusion criteria in both HAH and control groups using database queries against the EPR system.

Data on costs were obtained for the HAH service over FY23/24, covering all expenditures, including salaries, equipment, software licensing, capital expenditures, funding allocated for related CLCH services, and overheads.

HAH onboardings were classified as either AP, AD, or ESD. Admission prevention was defined for cases without ED or hospital admission within 48 h of onboarding; admission diversion was defined for cases admitted to the ED or hospital but discharged within 48 h of presentation; and ESD was defined where the duration of the inpatient admission was ≥48 h.

Inpatient escalation was defined as readmission to inpatient care during HAH (and within 24 h of discharge from HAH to allow for administrative uncertainty). Thirty-day readmission was defined as return to inpatient care within 30 days of discharge from the acute care episode (whether inpatient or HAH). Statistics for the total length of inpatient care over 90 days were measured from the initial presentation (i.e., the time of admission to the ED or hospital).

Inpatients declining HAH care were identified by a periodic review of operational worksheets and logbooks created by the patient screening team. Case records for all patients declining HAH care from the ED or the acute hospital were reviewed by one author (MS), who verified eligibility for VH care and categorised contemporaneously recorded reasons for declining based on screening and EPR documentation.

Data on deprivation were derived from the ONS Postcode File (OPF), census area definitions, and indices of multiple deprivation (IMD) datasets. Shape definitions for geographic figures were obtained from ONS census area shapefiles.

Data on quality-of-life indices were obtained for the purpose of service quality monitoring using repeated brief written and telephone EQ-5D-3L questionnaires at onboarding, discharge from HAH services, and at 1 month after offboarding. Additional data on patient satisfaction and experience were obtained at discharge via a bespoke questionnaire.^2^

ICD10 problem data were derived from clinical coding data. History items were imputed at each episode based on the presence or absence of specific ICD10 codes during that or any previous admission. Episode problem items were imputed at each episode based on the presence or absence of specific ICD10 codes during the specific episode alone. ICD10 code mapping to diagnostic concepts was based on ICD10 semantics and analysis of recorded ICD10 codes within the cohort.

Charlson comorbidity indices were calculated based on demographics and the presence or absence of specific ICD10 codes in the clinical history, based on the classifications established by Glasheen et al. (10).

Data on driving distances to hospital sites were derived from OpenStreetMap (11) (OSM, OpenStreetMap Foundation, Cambridge, UK) data and processed using the open source routing machine (OSRM) (12). Driving times and distances were calculated between the geometric centre of each postcode associated with BAM or HAH patients and the validated WGS84 coordinates of each hospital, and the hospital with the shortest estimated driving time was selected.

### Data analysis

Data were analysed using R software developed in house for the purpose of analysis, to ensure reproducibility and explicability of results. R (13) and RStudio (14) were used for development and execution of the analyses.

Data were imported from all sources and identical duplicate records removed. Where multiple demographic records unambiguously described a single patient, they were combined. To address duplicate care episode records, inconsistently identified episodes, or overlapping episodes, episode lists for each patient were generated, and by an iterative process, each pair of overlapping episodes was merged in chronological order where both episodes overlapped the other by at least 99%. Based on a series of heuristics derived from a manual review of outlying cases, patient reviews likely to represent incomplete, unsigned drafts were removed.

Data on preceding and following episodes were identified for each episode record, including the previous and next episodes of HAH and IP care, and used to categorise HAH admissions as AP, AD, or ESD. Where multiple HAH episodes began within 48 h of a single inpatient admission (such as where patients were under a shared care of multiple pathways), the single HAH longest episode was included.

Continuous data on time since last inpatient admission and number of inpatient admissions in the last year were converted to categorical data because of domain knowledge indicating that admission frequency was likely to be non-linearly related to clinical outcomes, and because of the observation that for many episodes in both control and treatment groups, the preceding episode was outside the visible lead-in period, which was at least 1 year.

Data from all sources were combined to a single record for each care episode, including BAM inpatient episodes for controls, or BAM inpatient episodes followed by an HAH episode in HAH patients. Data held by community care providers relating to assessments preceding admission were not available for merging. Additional parameters (including Charlson Comorbidity indices) were derived and exclusion criteria applied. Then, patient matching and statistical analysis were applied.

### Statistical analysis

#### Patient matching

Patient matching was centred on inpatient episodes preceding either HAH onboarding or discharge without HAH care. AD and ESD cohorts were matched to inpatient controls. AP patients were not matched but treated as a separate cohort for analysis.

Nearest-neighbour matching was performed using the MatchIt package (15) in R. Inpatient BAM care episodes for matching to HAH inpatient-source episodes were drawn from a pool of BAM patient care episodes. The selection criteria for the control episode pool included admission to the Emergency Department or a BAM inpatient setting between 1 December 2021 and 1 May 2024, with either the primary or secondary ICD10 problem code included in a selection of codes derived from inpatient episodes leading to AD or ESD HAH admissions. Episodes were excluded where the patient had previously been admitted to the HAH service. Control episodes matched to ESD patients were additionally required to have a length of stay of at least 2 days, reflecting the complementary requirement of the ESD case definition. Callipers, trimming, or other matching restrictions were not otherwise employed.

Matching without replacement was conducted separately for ESD and AD cohorts based on nearest-neighbour propensity score matching at 1:1 ratio. Propensity scores were estimated by generalised linear models. Matching criteria were age at admission, gender, simplified census ethnic group, a clinical grouping of the primary problem of the last inpatient admission, number of episodes in the last year, time since last hospitalisation, IMD score percentile, closest hospital, distance to closest hospital, Charlson comorbidity index, admission problem group, and medical history (including the presence of atrial fibrillation or flutter, ischaemic heart disease, heart failure, asthma, bronchiectasis, COPD, chronic pulmonary disease, tobacco usage, malignancy, dementia, peripheral vascular disease, cerebrovascular disease, diabetes mellitus, hypertension, chronic kidney disease, chronic liver disease, rheumatic conditions, and peptic ulcers).

The adequacy of matching and covariate balance was assessed by a review and comparison of matching characteristics between groups and by the generation of love plots, distribution plots, and calculation of mean and standardised mean differences using the “Cobalt” R package (16). A threshold of 0.10 for mean differences and that of 1.5 for variance ratios were selected, with a target of <0.05 for mean differences of important variables.

### Multivariate regression correction

Following matching, for each primary and secondary outcome, a linear or logistic regression model relating the outcome to the treatment group and all matching parameters was fit for a doubly robust estimation to minimise residual confounding. Coefficients and confidence intervals for the outcomes of interest were reported.

Full tables that included the remaining regression coefficients were also prepared as Supplementary Material. However, it should be noted that as these other variables have been used for matching, they are conditional upon the propensity score matching, which is likely to bias their relationships to non-outcome parameters. They should not be interpreted as estimates of population-level associations: their primary purpose in this context is to adjust for any residual confounding rather than to provide interpretable effect sizes.

The presence of several low-prevalence factors amongst the matching parameters complicated standard logistic regression, leading to a pseudo-complete separation in some variables. Firth's penalised logistic regression correction as implemented in the logistf R package (17) was employed for binary outcomes including 30-day readmissions and 90-day mortality.

### Quasi-experiment

A quasi-experimental comparator cohort was created using patients who were admitted to the inpatient setting, screened for HAH care according to usual clinical practice, but who declined the offers of HAH care for non-medical reasons. This cohort was compared directly against pooled inpatient-source HAH episodes (e.g., AD and ESD) for the principal outcome of inpatient length of stay, secondary outcome of total time in hospital over 90 days from initial presentation, safety outcomes of all-cause readmissions within 30 days, and all-cause mortality within 90 days.

### Comparisons

Continuous variables were compared by using the Mann–Whitney *U*-test, with confidence intervals calculated by the bias-corrected and accelerated bootstrap method employing at least 10,000 iterations. Fishers' exact test was used for categorical comparisons, and the Clopper–Pearson method was used for calculating confidence intervals for proportions.

Serial quality-of-life survey data were first assessed using Friedman's rank-sum test to identify items with significant differences over time, with *post hoc* testing with Conover's all-pairs comparisons test.

### Patient and public involvement

Design and preliminary results from the study were discussed during regular meetings of the Health and Care Partnership responsible for delivery of the HAH service, which included patient and public representatives.

Patient views regarding HAH care were actively solicited via serial surveys on quality-of-life indicators taken at admission to HAH, discharge from HAH, and 1 month after discharge from HAH; a patient experience survey was also taken at discharge.

Comments were also elicited from patients who refused HAH care for non-medical reasons, and qualitative summaries of patient views and concerns were prepared.

## Results

**Figure 1:**
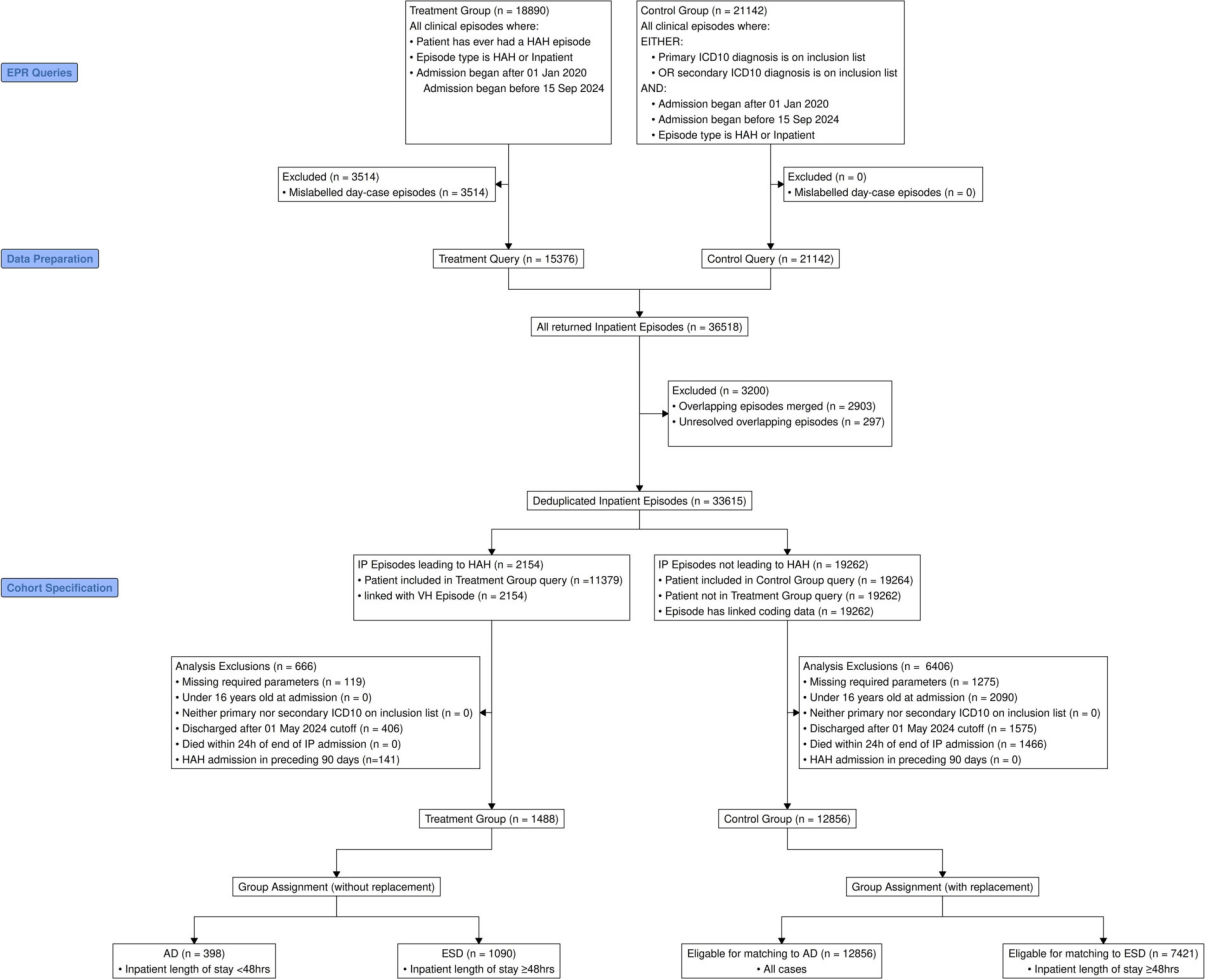
CONSORT diagram. Inclusion and exclusion criteria and cohort specification for AD and ESD groups. Additional information on classification of HAH cases is included in Supplementary Figure 3.

Between 22 December 2021 and 1 May 2024, 2,972 HAH patient care episodes were delivered for 2,406 distinct patients, with the break-up being 751 AP, 576 AD, 1,578 ESD, and 67 excluded episodes, principally ongoing cases (51). A total of 2,154 inpatient-sourced episodes were selected for matching, while by design, case matching was not performed in the AP cohort. A consort diagram was constructed to illustrate the reasons for exclusion (Figure 1).

In the inpatient-sourced HAH cohort, a total of 119 HAH episodes were excluded due to incomplete data, 406 HAH episodes due to insufficient follow-up period, and 141 HAH episodes due to previous HAH care within 90 days of the index inpatient episode. AD and ESD cohorts were matched with inpatient episodes as described above. After applying the exclusion criteria, 398 AD and 1,090 ESD episodes were used for matching and analysis.

A total of 21,142 control IP episodes were obtained based on ICD10 codes for primary or secondary admission diagnosis within the set of HAH index episode primary diagnoses over the study period. This yielded 19,262 episodes after de-duplication and merging of overlapping episodes, of which 1,275 episodes were excluded due to incomplete data, 2,090 episodes where the patient was under 16 years of age on admission, 1,575 episodes discharged after the 1 May 2024 cut-off date, and 1,466 episodes where the patient's recorded date of death was within 24 h of the discharge time. After applying the exclusion criteria, 12,856 control episodes remained for matching.

### Baseline demographics

**Figure 2:**
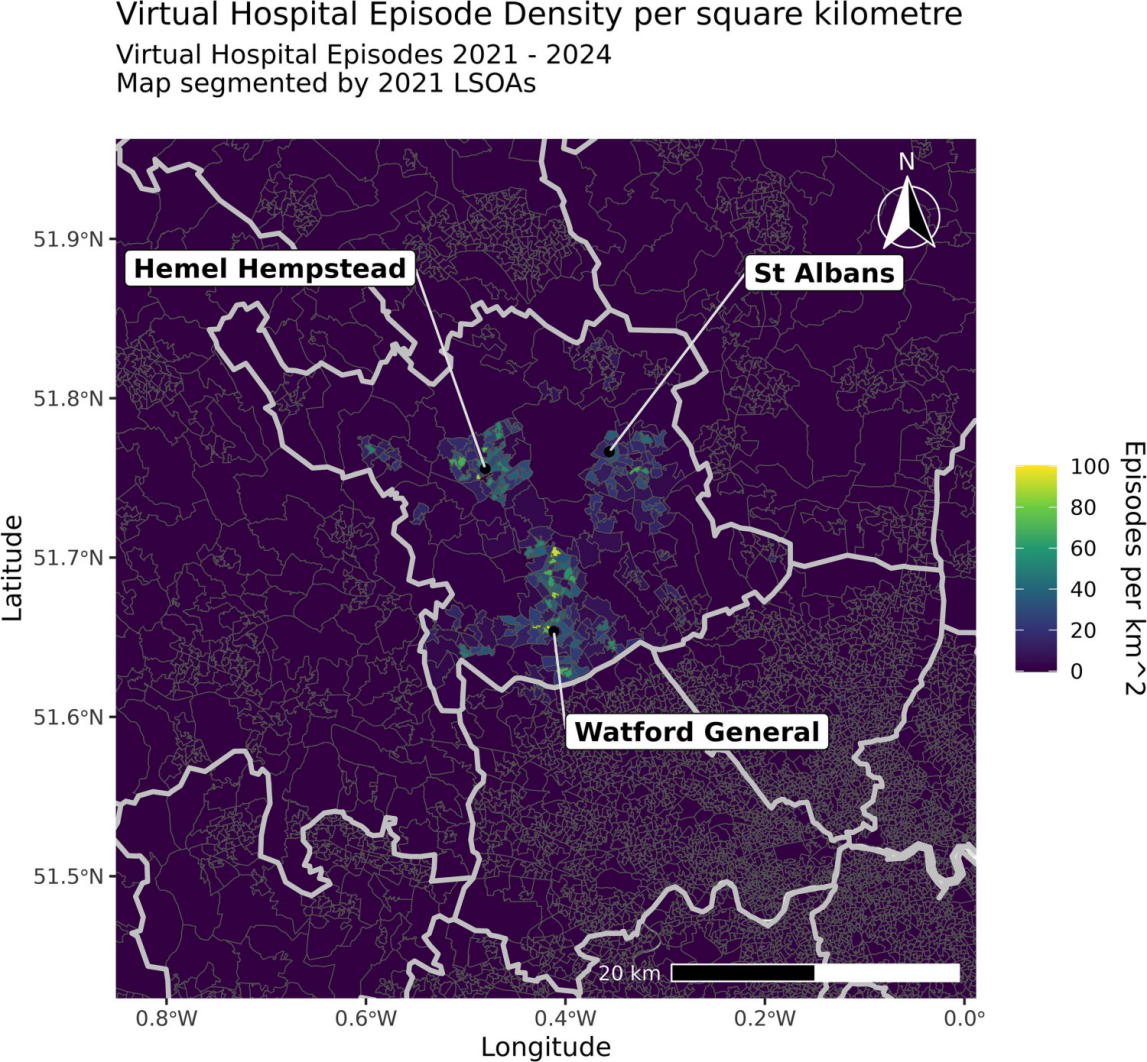
Virtual hospital episode density per square kilometre. Geospatial distribution of registered addresses of HAH patients demonstrates that most virtual hospital patients live within the West Herts Valley Sub-ICB Area, principally clustered in urban areas near hospital sites, with some patients located more peripherally. A comparison should be made with the population-adjusted rate (Supplementary Figure 1) and geographic distribution of deprivation (Supplementary Figure 2).

All three groups were broadly similar, with the mean age at presentation ranging between 60 and 70, roughly equal sex ratios (52% female), and similar predominantly White (87%–89%) ethnic makeup. Most patients (95%–96%) lived within the former West Hertfordshire Valley CCG area. The mean IMD percentile amongst HAH patients of 67 was above the national median of 50; however, this is broadly reflective of the average of the catchment area, with the subICB area having a mean population-weighted IMD percentile of 71, the 7th highest of 106 such areas across England.^3^ Baseline demographics, including geospatial information, presentation details, comorbidities, and frailty indications, are reported in Table 2 and Figure 2.

**Table 2:** Patient characteristics.

Characteristic	Community source	Inpatient source		Overall *N* = 2,915^a^
	AP *N* = 751^a^	AD *N* = 578^a^	ESD *N* = 1,586^a^	
Demographics				
Age at admission	70 (61, 81)	64 (52, 79)	69 (60, 80)	68 (59, 80)
Sex				
Female	373 (50%)	332 (57%)	800 (50%)	1,505 (52%)
Male	378 (50%)	246 (43%)	786 (50%)	1,410 (48%)
Census ethnicity group				
White	628 (89%)	473 (87%)	1,342 (89%)	2,443 (88%)
Asian or Asian British	44 (6.3%)	43 (7.9%)	113 (7.5%)	200 (7.2%)
Black or Black British	9 (1.3%)	11 (2.0%)	21 (1.4%)	41 (1.5%)
Chinese or other ethnic group	13 (1.9%)	10 (1.8%)	32 (2.1%)	55 (2.0%)
Mixed	8 (1.1%)	8 (1.5%)	7 (0.5%)	23 (0.8%)
Unknown or not stated	0 (0%)	0 (0%)	0 (0%)	0 (0%)
Geospatial details				
Local authority				
Dacorum	181 (24%)	134 (23%)	430 (27%)	745 (26%)
Hertsmere	71 (9.5%)	49 (8.5%)	135 (8.6%)	255 (8.8%)
St Albans	187 (25%)	121 (21%)	299 (19%)	607 (21%)
Three rivers	146 (19%)	115 (20%)	319 (20%)	580 (20%)
Watford	137 (18%)	135 (23%)	318 (20%)	590 (20%)
Other	27 (3.6%)	22 (3.8%)	72 (4.6%)	121 (4.2%)
Sub-ICB area				
West Herts Valley CCG	722 (96%)	555 (96%)	1,502 (95%)	2,779 (96%)
North West London ICB	7 (0.9%)	12 (2.1%)	39 (2.5%)	58 (2.0%)
Other	20 (2.7%)	9 (1.6%)	33 (2.1%)	62 (2.1%)
IMD percentile (lower is more deprived)	68 (47, 91)	65 (46, 88)	67 (47, 88)	67 (47, 89)
Nearest hospital				
Watford General Hospital	343 (46%)	302 (53%)	773 (49%)	1,418 (49%)
Hemel Hempsted Hospital	195 (26%)	145 (25%)	470 (30%)	810 (28%)
St Albans City Hospital	211 (28%)	128 (22%)	330 (21%)	669 (23%)
Driving distance to nearest hospital (miles)	6.9 (3.1, 7.6)	5.9 (2.8, 6.4)	7.0 (3.0, 6.9)	6.8 (3.0, 7.0)
Driving time to nearest hospital (mins)	9.9 (6.1, 11.5)	8.9 (5.5, 10.5)	9.8 (5.9, 11.1)	9.6 (5.8, 11.2)
Admission Details				
HAH Pathway				
ABC	242 (32%)	252 (44%)	611 (39%)	1,105 (38%)
ARI	105 (14%)	237 (41%)	476 (30%)	818 (28%)
HF	404 (54%)	89 (15%)	499 (31%)	992 (34%)
Length of HAH admission	12.3 (6.3, 15.1)	10.0 (5.3, 12.5)	10.2 (5.1, 13.2)	10.7 (5.3, 13.4)
Time from BAM discharge to HAH admission	NA (NA, NA)^b^	0.6 (0.4, 0.7)	0.5 (0.1, 0.7)	0.5 (0.2, 0.7)
Length of preceding admission	NA (NA, NA)^b^	0.9 (0.3, 1.4)	6.5 (3.2, 8.0)	5.0 (1.9, 6.7)
Primary inpatient problem				
Respiratory infection	0 (0%)	111 (19%)	351 (22%)	462 (16%)
Asthma exacerbation	0 (0%)	48 (8.3%)	105 (6.6%)	153 (5.2%)
Exacerbation of COPD or Bronchiectasis	0 (0%)	72 (12%)	214 (13%)	286 (9.8%)
Other pulmonary pathologies	0 (0%)	9 (1.6%)	28 (1.8%)	37 (1.3%)
Cardiac failure exacerbation	0 (0%)	31 (5.4%)	248 (16%)	279 (9.6%)
Atrial fibrillation	0 (0%)	9 (1.6%)	48 (3.0%)	57 (2.0%)
Ischaemic cardiac problems	0 (0%)	2 (0.3%)	36 (2.3%)	38 (1.3%)
Other cardiac pathologies	0 (0%)	5 (0.9%)	14 (0.9%)	19 (0.7%)
Sepsis	0 (0%)	0 (0%)	8 (0.5%)	8 (0.3%)
Other infection	0 (0%)	1 (0.2%)	12 (0.8%)	13 (0.4%)
Not recorded	751 (100%)	290 (50%)	522 (33%)	1,563 (54%)
Not categorised	0 (0%)	0 (0%)	0 (0%)	0 (0%)
Frailty indicators				
Type of residence^c^				
Private residence	727 (97%)	561 (97%)	1,522 (96%)	2,810 (96%)
Supported or retirement home	6 (0.8%)	8 (1.4%)	13 (0.8%)	27 (0.9%)
Residential and nursing home	18 (2.4%)	8 (1.4%)	49 (3.1%)	75 (2.6%)
Other (including temporary, shelter, or mental health facility)	0 (0%)	1 (0.2%)	2 (0.1%)	3 (0.1%)
Charlson Comorbidity Index	3.51 (2.00, 5.00)^d^	3.18 (1.00, 4.00)^e^	4.00 (2.00, 5.00)	3.74 (2.00, 5.00)
Problems recorded on most recent coding	4.8 (0.0, 9.0)^d^	5.2 (0.0, 9.0)^e^	8.0 (0.0, 13.0)	6.8 (0.0, 11.0)
Total unique problems to most recent coding	7 (0, 11)^d^	7 (0, 10)^e^	10 (0, 15)	9 (0, 14)
Number of hospital visits in preceding year	1.5 (0.0, 2.0)^f^	1.3 (0.0, 2.0)^e^	1.4 (0.0, 2.0)	1.4 (0.0, 2.0)
Total time in hospital in preceding year (days)	6.2 (0.0, 7.2)^f^	4.3 (0.0, 2.9)^e^	6.1 (0.0, 6.1)	5.8 (0.0, 5.7)
Number of hospital visits in following 90 days	0.6 (0.0, 1.0)^f^	1.5 (1.0, 2.0)^e^	1.6 (1.0, 2.0)	1.3 (1.0, 2.0)
Total time in hospital in following 90 days (days)	3.3 (0.0, 1.3)^f^	3.0 (0.5, 2.0)^e^	10.4 (3.8, 12.0)	7.1 (0.8, 8.7)
Medical History Coding Items				
Diabetes	72 (13%)^d^	75 (15%)^e^	275 (17%)	422 (16%)
Hypertension	162 (30%)^d^	166 (32%)^e^	704 (44%)	1,032 (39%)
Asthma	61 (11%)^d^	117 (23%)^e^	289 (18%)	467 (18%)
Bronchiectasis	43 (8.0%)^d^	37 (7.2%)^e^	140 (8.8%)	220 (8.4%)
COPD	108 (20%)^d^	134 (26%)^e^	480 (30%)	722 (27%)
Chronic pulmonary disease	173 (32%)^d^	241 (47%)^e^	769 (48%)	1,183 (45%)
Recorded tobacco use	39 (7.3%)^d^	69 (13%)^e^	209 (13%)	317 (12%)
Atrial fibrillation	109 (20%)^d^	77 (15%)^e^	429 (27%)	615 (23%)
Myocardial infarction	38 (7.1%)^d^	32 (6.2%)^e^	164 (10%)	234 (8.9%)
Heart failure	129 (24%)^d^	85 (17%)^e^	546 (34%)	760 (29%)
Peripheral vascular disease	23 (4.3%)^d^	13 (2.5%)^e^	86 (5.4%)	122 (4.6%)
Cerebrovascular disease	9 (1.7%)^d^	5 (1.0%)^e^	58 (3.7%)	72 (2.7%)
Dementia	6 (1.1%)^d^	7 (1.4%)^e^	19 (1.2%)	32 (1.2%)
Rheumatoid disease	22 (4.1%)^d^	15 (2.9%)^e^	109 (6.9%)	146 (5.5%)
Peptic ulcer disease	2 (0.4%)^d^	1 (0.2%)^e^	9 (0.6%)	12 (0.5%)
Mild liver disease	17 (3.2%)^d^	18 (3.5%)^e^	79 (5.0%)	114 (4.3%)
Moderate or severe liver disease	3 (0.6%)^d^	2 (0.4%)^e^	5 (0.3%)	10 (0.4%)
Mild or moderate renal disease	36 (6.7%)^d^	35 (6.8%)^e^	168 (11%)	239 (9.1%)
Severe renal disease	2 (0.4%)^d^	2 (0.4%)^e^	11 (0.7%)	15 (0.6%)
Malignancy without metastases	12 (2.2%)^d^	22 (4.3%)^e^	102 (6.4%)	136 (5.2%)
Malignancy with metastases	1 (0.2%)^d^	6 (1.2%)^e^	34 (2.1%)	41 (1.6%)

Baseline characteristics of HAH patients by source and overall, including demography, geography, indicators of socioeconomic status, health, and frailty, and details of BAM care leading to HAH presentation. Parameters are broadly similar across groups, although ESD patients appear to be generally older, measure slightly higher on markers of frailty, and live slightly further away from the hospital compared with either AD or AP patients, and patterns are apparent in admission sources by the HAH pathway.

^a^ Mean (Q1, Q3); *n* (%).

^b^ No preceding clinically linked episode.

^c^ Last recorded address at time of data retrieval; may differ from address during care episode.

^d^ Excluding 228/751 (30%) of cases lacking clinical coding.

^e^ Excluding 58/578 (10%) of cases lacking clinical coding.

^f^ Index time at HAH presentation rather than hospital presentation.

Respiratory indications, including asthma, COPD, respiratory tract infections, and other pulmonary pathologies, were the largest single group of presenting diagnoses, accounting for 69% (938/1,352) of recorded, categorised principal diagnoses. In contrast, 29% (393/1,352) of recorded, categorised presentations held a cardiac primary diagnosis, including AF, heart failure, ischaemic, and other cardiac problem groups. However, limited formal diagnosis details are available for AP patients due to their definitional lack of IP care for clinical coding, as was the case for some (27%, 137/578) AD patients who were discharged from the ED to HAH without IP assessment. This makes precise comparisons of presenting diagnosis across pathways challenging and increases the relative weight of ESD episodes in these data.

There were some notable differences: AD patients were broadly younger than the other groups [64 years vs. 70 years (AP) vs. 69 years (ESD)], slightly more likely to be male (57% vs. 50% in both AP and ESD), more likely to be admitted under the ARI pathway [44% vs. 14% (AP) vs. 30% (ESD)], and less likely to be admitted under HF [15% vs. 54% (AP) vs. 31% (ESD)]. While patients were admitted to the three pathways in roughly equal numbers under the ESD pathway, AP cases were dominated by HF patients (54%), with a number of these referrals being made directly from the Rapid Access Heart Failure Clinic service.

Rates of significant comorbidities were considerable across all three pathways; for instance, 20%–30% of patients suffered from COPD, 15%–27% from AF, and 13%–17% from diabetes. ESD patients tended to demonstrate the greatest clinical complexity, with greater Charlson Comorbidity indices [4.0 vs. 3.5 (AP) vs. 3.2 (AD)], greater numbers of notable past medical history items [8 vs. 4.8 (AP) vs. 5.2 (AD)], and greater prevalence of all conditions of interest except asthma, severe liver disease, recorded tobacco use, and dementia. For instance, chronic lung diseases had a prevalence rate of 48% in the ESD cohort, compared with 47% in AD and only 32% in AP, while heart failure affected 34% of ESD patients vs. 24% of AP and 17% of AD patients.

### HAH length of stay

Length of HAH stay was approximately 10 days in inpatient sourced admissions: 10.0 days (median 8.0, IQR 5.3–12.5, max 69.4) for AD patients, and 10.2 days (median 8.2, IQR 5.1–13.2, max 63.1) within the ESD cohort. The AP cohort averaged 12.3 days (median 10.4, IQR 6.3–15.1, max 140), possibly reflecting a “head start” provided by initial hospital assessment in the other groups.

### Matching

**Figure 3:**
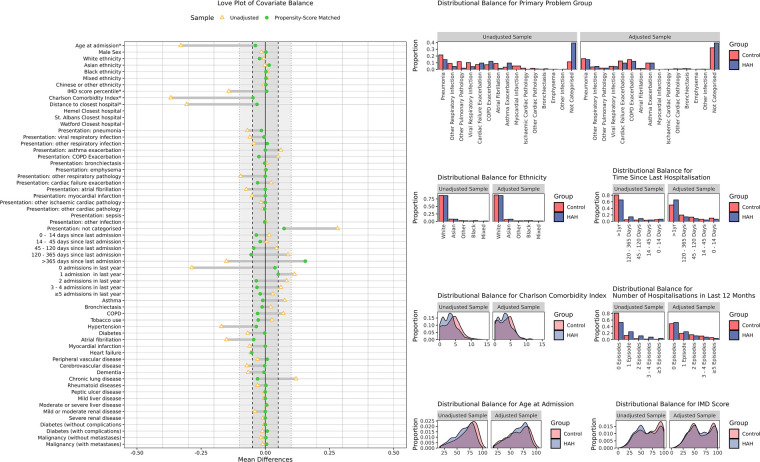
Covariate balance in the AD cohort. Covariate balance improved following propensity score matching, with all but one measures falling within the pre-specified ±0.1 range for mean differences and the majority falling within the desirable ±0.05 range. A graphical review of selected non-binary parameters demonstrates good distributional balance following matching.

**Figure 4:**
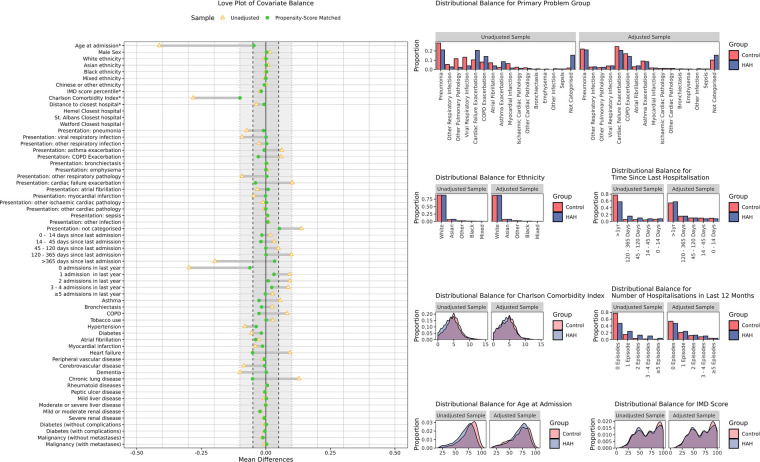
Covariate balance in the ESD cohort. Covariate balance improved following propensity score matching, with all measures falling within the pre-specified ±0.1 range for mean differences and the majority falling within the desirable ±0.05 range. A graphical review of selected non-binary parameters demonstrates good distributional balance following matching.

Matching was conducted separately for the AD and ESD cohorts, on the basis of the inpatient source episodes. Controls for both AD and ESD were drawn from the same pool of IP episodes; however, within each group, matching was without replacement. It was determined that standardised mean differences of <0.1 were acceptable, with ≤0.05 preferable for important parameters. A threshold of 1.5× for variance ratio was selected (e.g., 0.67–1.5). Matching quality information is presented in Tables 3, 4; Figures 3, 4.

**Table 3:** Balance in matching for AD cohort.

Characteristic	Balance indicators for propensity score matching of the AD cohort						
	Type	Diff Un	V.Ratio Un	Diff. Adj	M. Threshold	V.Ratio Adj	V.Threshold
Charlson Comorbidity Index^a^	Contin.	−0.37	0.81	−0.05	Balanced, <0.1	1.00	Balanced, <1.5
Days since last admission							
>365	Binary	−0.15	–	0.16	Not Balanced, >0.1	–	
120–365	Binary	0.09	–	−0.06	Balanced, <0.1	–	
45–120	Binary	0.04	–	−0.05	Balanced, <0.1	–	
14–45	Binary	0.01	–	−0.02	Balanced, <0.1	–	
0–14	Binary	0.01	–	−0.04	Balanced, <0.1	–	
Admissions in last year							
0	Binary	−0.29	–	0.04	Balanced, <0.1	–	
1	Binary	0.11	–	0.05	Balanced, <0.1	–	
2	Binary	0.08	–	−0.04	Balanced, <0.1	–	
3–4	Binary	0.06	–	−0.03	Balanced, <0.1	–	
≥5	Binary	0.03	–	−0.02	Balanced, <0.1	–	
Age at admission^a^	Contin.	−0.33	0.93	−0.04	Balanced, <0.1	0.80	Balanced, <1.5
Male sex	Binary	−0.02	–	0.00	Balanced, <0.1	–	
Ethnicity							
White	Binary	−0.01	–	−0.02	Balanced, <0.1	–	
Asian	Binary	0.00	–	0.02	Balanced, <0.1	–	
Chinese or other	Binary	−0.01	–	0.00	Balanced, <0.1	–	
Black	Binary	0.01	–	0.00	Balanced, <0.1	–	
Mixed	Binary	0.00	–	0.00	Balanced, <0.1	–	
IMD score percentile^a^	Contin.	−0.14	1.01	0.00	Balanced, <0.1	1.01	Balanced, <1.5
Closest Hospital							
Watford General	Binary	−0.02	–	0.02	Balanced, <0.1	–	
Hempstead Hempstead	Binary	0.00	–	0.01	Balanced, <0.1	–	
St Albans City	Binary	0.02	–	−0.03	Balanced, <0.1	–	
Distance to closest hospital^a^	Contin.	−0.31	0.13	−0.03	Balanced, <0.1	0.40	Not balanced, >1.5
Diabetes	Binary	−0.07	–	0.00	Balanced, <0.1	–	
Hypertension	Binary	−0.17	–	−0.04	Balanced, <0.1	–	
Asthma	Binary	0.08	–	−0.01	Balanced, <0.1	–	
Bronchiectasis	Binary	0.02	–	−0.01	Balanced, <0.1	–	
COPD	Binary	0.07	–	−0.03	Balanced, <0.1	–	
Tobacco use	Binary	0.03	–	−0.03	Balanced, <0.1	–	
Atrial fibrillation	Binary	−0.15	–	−0.05	Balanced, <0.1	–	
Myocardial infarction	Binary	−0.06	–	0.00	Balanced, <0.1	–	
Heart failure	Binary	−0.06	–	−0.05	Balanced, <0.1	–	
Peripheral vascular disease	Binary	−0.03	–	0.01	Balanced, <0.1	–	
Cerebrovascular disease	Binary	−0.07	–	0.00	Balanced, <0.1	–	
Dementia	Binary	−0.07	–	−0.01	Balanced, <0.1	–	
Chronic lung disease	Binary	0.12	–	−0.03	Balanced, <0.1	–	
Rheumatoid diseases	Binary	−0.03	–	0.00	Balanced, <0.1	–	
Peptic ulcer disease	Binary	0.00	–	0.00	Balanced, <0.1	–	
Liver Disease							
Mild	Binary	−0.01	–	0.00	Balanced, <0.1	–	
Moderate or severe	Binary	0.01	–	0.01	Balanced, <0.1	–	
Renal Disease							
Mild or moderate	Binary	−0.04	–	–	Balanced, <0.1	–	
Severe	Binary	−0.01	–	–	Balanced, <0.1	–	
Diabetes							
Without complications	Binary	0.00	–	–	Balanced, <0.1	–	
With complications	Binary	−0.01	–	–	Balanced, <0.1	–	
Malignancy							
Without metastases	Binary	−0.02	–	0.00	Balanced, <0.1	–	
With metastases	Binary	−0.01	–	0.01	Balanced, <0.1	–	
Presentation							
Pneumonia	Binary	−0.07	–	−0.02	Balanced, <0.1	–	
Other respiratory infections	Binary	−0.04	–	0.01	Balanced, <0.1	–	
Other respiratory pathologies	Binary	−0.10	–	0.00	Balanced, <0.1	–	
Viral respiratory infection	Binary	−0.06	–	−0.01	Balanced, <0.1	–	
Cardiac failure exacerbation	Binary	0.02	–	−0.03	Balanced, <0.1	–	
COPD Exacerbation	Binary	0.05	–	−0.03	Balanced, <0.1	–	
Atrial fibrillation	Binary	−0.07	–	0.00	Balanced, <0.1	–	
Asthma exacerbation	Binary	0.06	–	0.00	Balanced, <0.1	–	
Myocardial infarction	Binary	−0.05	–	0.00	Balanced, <0.1	–	
Other ischaemic cardiac pathologies	Binary	−0.02	–	0.00	Balanced, <0.1	–	
Other cardiac pathologies	Binary	−0.01	–	0.00	Balanced, <0.1	–	
Bronchiectasis	Binary	0.01	–	0.00	Balanced, <0.1	–	
Emphysema	Binary	0.00	–	0.00	Balanced, <0.1	–	
Other infections	Binary	0.00	–	0.00	Balanced, <0.1	–	
Not categorised	Binary	0.28	–	0.07	Balanced, <0.1	–	

Balance indicators for propensity score matching in AD (6a) and ESD (6b) cohorts. Indicators demonstrated good matching achieved across almost all parameters, with a small amount of residual imbalance in the proportion of AD HAH patients who had not been admitted to BAM hospital in the last year (fewer before and greater after matching), and distance to closest BAM hospital in the AD cohort (slightly nearer), and in the Charlson Comorbidity Index in the ESD group (which was slightly higher for ESD HAH patients). This should be reviewed alongside love plots and distributions of the relevant parameters (see figure).

^a^ Asterisks indicate standardised means.

**Table 4:** Balance in matching for the ESD cohort.

Characteristic	Balance indicators for propensity score matching of the ESD cohort						
	Type	Diff Un	V.Ratio Un	Diff. Adj	M.Threshold	V.Ratio Adj	V.Threshold
Charlson Comorbidity Index^a^	Contin.	−0.28	1.01	−0.10	Not balanced, >0.1	0.95	Balanced, <1.5
Days since last admission							
>365	Binary	−0.20	–	0.03	Balanced, <0.1	–	
120–365	Binary	0.10	–	0.00	Balanced, <0.1	–	
45–120	Binary	0.05	–	0.00	Balanced, <0.1	–	
14–45	Binary	0.03	–	−0.02	Balanced, <0.1	–	
0–14	Binary	0.02	–	−0.02	Balanced, <0.1	–	
Admissions in last year							
0	Binary	−0.30	–	−0.06	Balanced, <0.1	–	
1	Binary	0.09	–	0.03	Balanced, <0.1	–	
2	Binary	0.09	–	0.01	Balanced, <0.1	–	
3–4	Binary	0.09	–	0.02	Balanced, <0.1	–	
≥5	Binary	0.03	–	0.00	Balanced, <0.1	–	
Age at admission^a^	Contin.	−0.41	1.10	−0.05	Balanced, <0.1	0.78	Balanced, <1.5
Male sex	Binary	0.02	–	0.00	Balanced, <0.1	–	
Ethnicity							
White	Binary	−0.01	–	0.00	Balanced, <0.1	–	
Asian	Binary	0.01	–	0.00	Balanced, <0.1	–	
Chinese or other	Binary	0.00	–	−0.01	Balanced, <0.1	–	
Black	Binary	0.00	–	0.00	Balanced, <0.1	–	
Mixed	Binary	0.00	–	0.00	Balanced, <0.1	–	
IMD score percentile^a^	Contin.	−0.02	0.95	−0.02	Balanced, <0.1	0.99	Balanced, <1.5
Closest hospital							
Watford General	Binary	−0.01	–	0.02	Balanced, <0.1	–	
Hempstead Hempstead	Binary	0.03	–	0.00	Balanced, <0.1	–	
St Albans City	Binary	−0.01	–	−0.01	Balanced, <0.1	–	
Distance to closest hospital^a^	Contin.	−0.04	0.87	−0.01	Balanced, <0.1	1.11	Balanced, <1.5
Diabetes	Binary	−0.06	–	−0.02	Balanced, <0.1	–	
Hypertension	Binary	−0.08	–	−0.04	Balanced, <0.1	–	
Asthma	Binary	0.06	–	−0.03	Balanced, <0.1	–	
Bronchiectasis	Binary	0.02	–	−0.02	Balanced, <0.1	–	
COPD	Binary	0.08	–	−0.03	Balanced, <0.1	–	
Tobacco use	Binary	0.03	–	0.00	Balanced, <0.1	–	
Atrial fibrillation	Binary	−0.03	–	−0.04	Balanced, <0.1	–	
Myocardial infarction	Binary	−0.04	–	−0.01	Balanced, <0.1	–	
Heart failure	Binary	0.09	–	−0.05	Balanced, <0.1	–	
Peripheral vascular disease	Binary	−0.01	–	−0.01	Balanced, <0.1	–	
Cerebrovascular disease	Binary	−0.09	–	0.00	Balanced, <0.1	–	
Dementia	Binary	−0.10	–	0.00	Balanced, <0.1	–	
Chronic lung disease	Binary	0.13	–	−0.05	Balanced, <0.1	–	
Rheumatoid diseases	Binary	0.00	–	0.01	Balanced, <0.1	–	
Peptic ulcer disease	Binary	0.00	–	0.00	Balanced, <0.1	–	
Liver disease							
Mild	Binary	−0.01	–	0.00	Balanced, <0.1	–	
Moderate or severe	Binary	0.00	–	0.00	Balanced, <0.1	–	
Renal disease							
Mild or moderate	Binary	−0.02	–	−0.02	Balanced, <0.1	–	
Severe	Binary	−0.01	–	−0.01	Balanced, <0.1	–	
Diabetes							
Without complications	Binary	0.00	–	0.00	Balanced, <0.1	–	
With complications	Binary	−0.01	–	0.00	Balanced, <0.1	–	
Malignancy							
Without metastases	Binary	−0.01	–	−0.01	Balanced, <0.1	–	
With metastases	Binary	−0.01	–	0.00	Balanced, <0.1	–	
Presentation							
Pneumonia	Binary	−0.07	–	−0.01	Balanced, <0.1	–	
Other respiratory infections	Binary	−0.03	–	0.00	Balanced, <0.1	–	
Other respiratory pathologies	Binary	−0.09	–	0.00	Balanced, <0.1	–	
Viral respiratory infection	Binary	−0.09	–	0.00	Balanced, <0.1	–	
Cardiac failure exacerbation	Binary	0.10	–	−0.04	Balanced, <0.1	–	
COPD Exacerbation	Binary	0.06	–	−0.03	Balanced, <0.1	–	
Atrial fibrillation	Binary	−0.03	–	0.01	Balanced, <0.1	–	
Asthma exacerbation	Binary	0.06	–	−0.01	Balanced, <0.1	–	
Myocardial infarction	Binary	−0.05	–	0.00	Balanced, <0.1	–	
Other ischaemic cardiac pathologies	Binary	−0.01	–	0.00	Balanced, <0.1	–	
Other cardiac pathologies	Binary	−0.01	–	0.00	Balanced, <0.1	–	
Bronchiectasis	Binary	0.00	–	0.00	Balanced, <0.1	–	
Emphysema	Binary	0.00	–	0.00	Balanced, <0.1	–	
Other infections	Binary	0.01	–	0.01	Balanced, <0.1	–	
Sepsis	Binary	0.01	–	0.01	Balanced, <0.1	–	
Not categorised	Binary	0.14	–	0.05	Balanced, <0.1	–	

^a^ Asterisks indicate standardised means.

Indicators of matching quality within the AD cohort were generally good, with all but one of 61 matching parameters achieving an adjusted difference of ≤0.1 (Table 3; Figure 3). The sole exception was the proportion of patients for whom the preceding hospital admission was over a year ago, which was greater in controls prior to matching and lower in controls after matching (66% vs. 81% before matching in HAH and control patients, respectively; 66% vs. 50% after matching). Of the remaining matching parameters, all demographic factors (age, sex, ethnicity), all specific conditions (e.g., asthma, heart failure, and dementia), and all presenting problem groups, except “not categorised,” achieved a closely matched mean difference of ≤0.05. Variance ratios of continuous parameters were well within thresholds (1.0, 0.8, and 1.0 for the Charlson Comorbidity Index, IMD socioeconomic status, and age, respectively), except for distance to nearest hospital, which had a broader variance of 0.40, improved by matching from 0.13. This was acceptable given the limited expected association with propensity and outcomes and good matching across geospatial covariates.

The matching performance was excellent in the ESD cohort. All parameters met the SMD ≤0.1 threshold, with most of them also reaching the ≤0.05 threshold—with the exceptions of the Charlson Comorbidity Index (0.10) and the proportion with no admissions in the last year (0.06) (Table 4, Figure 7). The mean and standard deviation of the CCI was similar in both ESD and control cohorts both before and after matching (pre-matching: 4.8 ± 2.3 vs. 4.2 ± 2.3; postmatching: 4.4 ± 2.4 vs. 4.2 ± 2.3) All continuous variables met the Variance Ratio <1.5 threshold, with the greatest difference in age at admission (0.78).

Noting the potential for control episodes to be selected for both ESD and AD matching, selected controls were assessed and a total of 102 control episodes were matched to both cohort times. There was otherwise no sampling with replacement and no scope for episodes to be otherwise matched to a greater number of HAH episodes.

The overall matching performance was judged good, with excellent matching achieved in the ESD cohort and acceptable matching in the smaller AD cohort.

### Inpatient length of stay

Principal outcomes are presented in Table 5. Within the AD cohort, univariate comparisons against the AD-matched control cohort demonstrated a 4.36-day reduction in mean length of stay (LOS) from 5.28 to 0.92 days (95% CI 3.51–5.31 days, *p* < 2 × 10^−8^). Regression adjustment yielded a doubly robust estimate of 3.92-day savings (95% CI 3.04 −4.81, *p* < 3 × 10^−17^).

**Table 5:** Principal results and safety outcomes.

Measure	Cohort	Unadjusted	Regression-adjusted
Principal outcomes		difference (95% CI, treatment vs. control, *p*-value)	difference (95% CI, *p*-value)
Reduction in BAM inpatient length of stay	AD	4.36 days (3.51–5.31, 0.92 vs. 5.28, *p* < 2 × 10^−8^)	3.92 days (3.04–4.81, *p* < 3 × 10^−17^)
	ESD	3.08 days (2.46–3.75, 6.24 vs. 9.32, *p* < 2 × 10^−15^)	2.98 days (2.34–3.63, *p* < 2 × 10^−19^)
	Overall	3.42 days (2.89–3.98, 4.82 vs. 8.24, *p* < 1 × 10^−29^)	3.13 days (2.60–3.67, *p* < 1 × 10^−29^)
	vs. HAH Decliners	3.92 days (2.25–5.90, 4.82 vs. 8.74, *p* < 3 × 10^−6^)	–
Reduction in calendar days with any BAM hospital care in 90 days following initial presentation	AD	4.79 days (3.39–6.22, 4.22 vs. 9.00, *p* < 1 × 10^−8^)	3.97 days (2.64–5.30, *p* < 8 × 10^−9^)
	ESD	2.25 days (1.29–3.24, 11.07 vs. 13.32, *p* < 3 × 10^−6^)	2.20 days (1.25–3.16, *p* < 7 × 10^−6^)
	Overall	2.93 days (2.09–3.78, 9.24 vs. 12.17, *p* < 5 × 10^−14^)	2.54 days (1.75–3.33, *p* < 4 × 10^−10^)
Reduction in the total duration of BAM hospital admissions in 90 days following initial presentation	AD	4.82 days (3.50–6.17, 2.90 vs. 7.73, *p* < 1 × 10^−4^)	4.08 days (2.81–5.36, *p* < 6 × 10^−10^)
	ESD	2.34 days (1.43–3.30, 9.70 vs. 12.05, *p* < 4 × 10^−7^)	2.29 days (1.37–3.21, *p* < 2 × 10^−6^)
	Overall	3.01 days (2.21–3.83, 7.88—10.89, *p* < 8 × 10^−16^)	2.64 days (1.87–3.40, *p* < 2 × 10^−11^)
**Safety outcomes**		**OR (95% CI, treatment vs. control, *p*-value)**	**OR (95% CI, *p*-value)**
Risk of readmission to the BAM hospital at 30 days (any cause)	AD	OR 1.11 (0.76–1.64, 18% vs. 16%, *p* < 7 × 10^−1^, ns)	OR 0.41 (0.24–0.68, *p* < 5 × 10^−4^)
	ESD	OR 1.54 (1.22–1.95, 20% vs. 14%, *p* < 2 × 10^−4^)	OR 0.62 (0.46–0.84, *p* < 2 × 10^−3^)
	Combined	OR 1.41 (1.16–1.72, 19% vs. 15%, *p* < 6 × 10^−4^)	OR 0.55 (0.42–0.70, *p* < 3 × 10^−6^)
	vs. HAH Decliners	OR 0.19 (0.11–0.32, 8% vs. 32%, *p* < 3 × 10^−9^)	–
Risk of escalation of care from HAH to BAM (any cause)	AD	14% (55/398, 95% CI 11%–18%)	–
	ESD	14% (157/1,090, 95% CI 12%–17%)	–
	Combined	14% (212/1,488, 95% CI 12%–16%)	–
Risk of death at 90 days (any cause)	AD	OR 0.34 (0.22–0.51, 10% vs. 24%, *p* < 8 × 10^−8^)	OR 0.34 (0.20–0.55, *p* < 6 × 10^−6^)
	ESD	OR 0.45 (0.36–0.55, 17% vs. 31%, *p* < 5 × 10^−15^)	OR 0.45 (0.35–0.56, *p* < 7 × 10^−11^)
	Combined	OR 0.42 (0.35–0.51, 15% vs. 29%, *p* < 4 × 10^−21^)	OR 0.43 (0.35–0.53, *p* < 3 × 10^−16^)
	vs. HAH Decliners	OR 0.69 (0.39–1.28, 15% vs. 20%, *p* < 3 × 10^−1^, ns)	–

Both unadjusted and doubly robust, regression-adjusted results demonstrate a marked reduction in BAM length of stay, which is recapitulated in the pseudo-experimental cohort, and preserved at 90 days. While HAH patients were readmitted more frequently than matched controls, HAH was protective against all-cause readmission after controlling for residual imbalance in patient characteristics, which is in agreement with the pseudo-experimental cohort. Intriguingly, risk of death at 90 days was lower across all three groups, although this finding must be interpreted cautiously.

The results in the ESD cohort were similar, with univariate comparisons showing a 3.08-day reduction from 9.32 to 6.24 days (95% CI 2.46–3.75, *p* < 2 × 10^−15^) and a regression-adjusted doubly robust estimated reduction in length of stay of 2.98 days (95% CI 2.34–3.63, *p* < 2 × 10^−19^).

When pooling data from both cohorts, the mean regression-adjusted estimated reduction in length of stay was 3.13 days (95% CI 2.60–3.67, *p* < 1 × 10^−29^).

An analysis comparing LOS data for AD and HAH patients with a pseudo-experimental control group of patients who met HAH criteria but declined onboarding showed an overall mean difference in length of stay pooled across both ESD and AD groups of 3.92 days (4.82 vs. 8.74; 95% CI 2.25–5.90 days; *p* < 3 × 10^−6^, *n* = 1,573 including 85 refusers).

In the AP cohort, inpatient LOS was, by definition, zero days; this group is not directly comparable to the other two groups due to differences in available data for outpatients, but if it is assumed in line with the service design that these patients are similar to the other two cohorts and would otherwise require hospitalisation, it would be reasonable to take into account the case number weighted average length of stay of the AD and ESD control cohorts (i.e., 8.2 days).

### Cost–benefit analysis

The cost of HAH care was determined by amortising the total programme expenditure for the ABC, ARI, and HF HAH pathways over FY23/24 of £1,904,831 by the total number of bed days of HAH care delivered by the service over that period, 16,072.75 bed days. This yielded a cost per bed day of £118.51.

The cost of inpatient care was obtained from the finance department at the West Herts Teaching Hospitals Trust, with £569 reflecting fully absorbed costs per hospital bed day.

On the basis of a 3.92 mean inpatient bed-day saving per AD patient (576 patients), a 2.98 bed-day saving per ESD patient (1,578 patients), and an 8.2 bed-day saving per AP patient (751 patients), a total saving of 13,118.56 bed days is calculated with a gross value of £7,464,461.

Given a cost of £118.51 per HAH bed day, the above patient counts, and the mean lengths of HAH care across the whole patient group of 12.2, 10.0, and 10.2 days in AP, AD, and ESD respectively, the cost of the HAH care intervention is calculated as £3,675,919, producing a net cost benefit of £3.79 m over the 33 months of the evaluation between December 2021 and September 2024. Re-expressed in terms of individual episodes, this amounts to a net benefit of £3,219.98 per AP episode, £1,045.38 per AD episode, and £486.82 per ESD episode.

### Patient experience and acceptability

**Figure 5:**
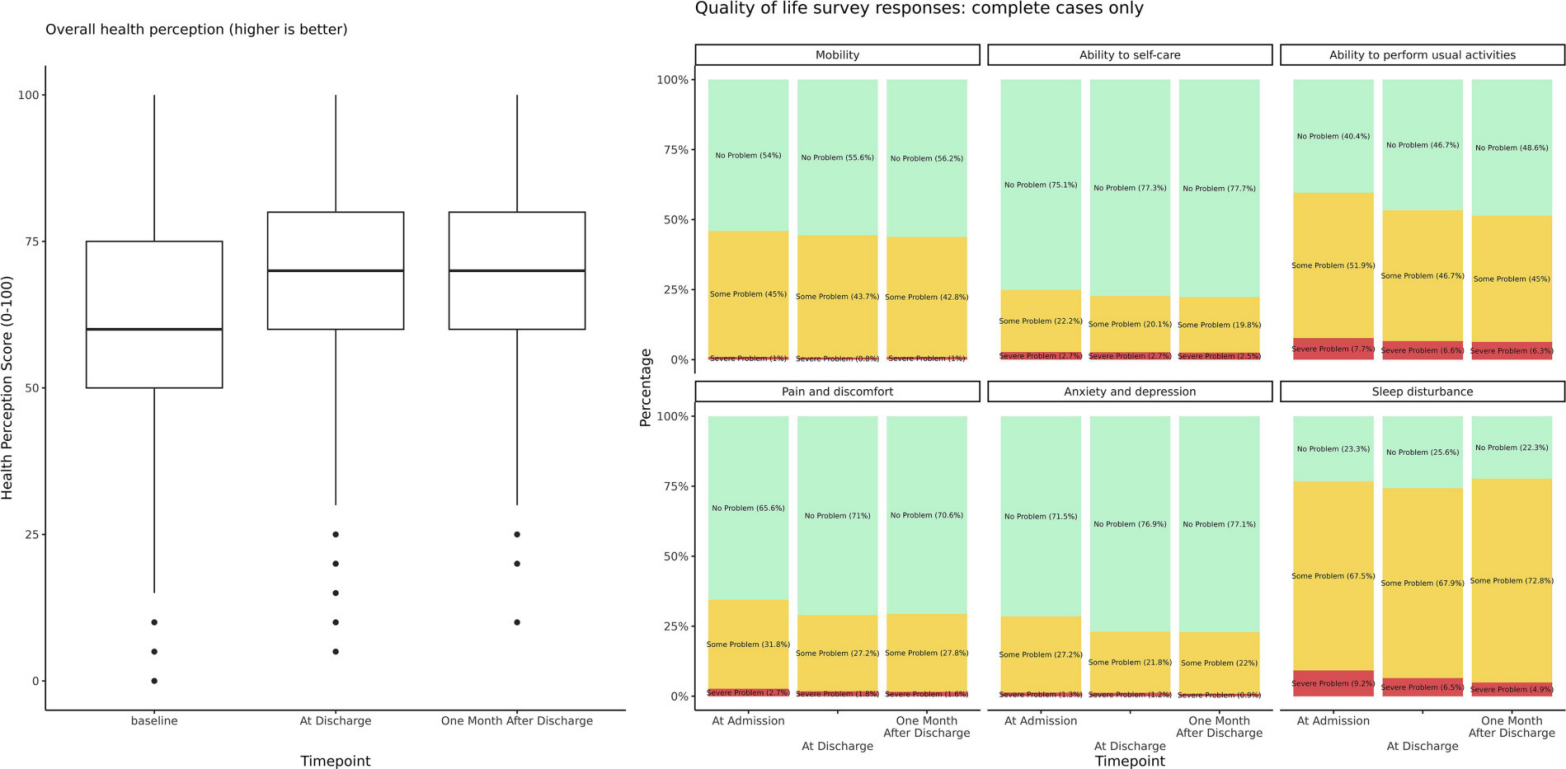
Quality-of-life survey responses: Complete cases only. Overall health perception scores, as well as patient-reported measures of mobility, self-care, ability to perform usual activities, pain and discomfort, anxiety and depression, and sleep improved with admission to HAH, with improvements persisting at one month after HAH discharge. These results reflect patients who responded at all three time points only.

**Figure 6:**
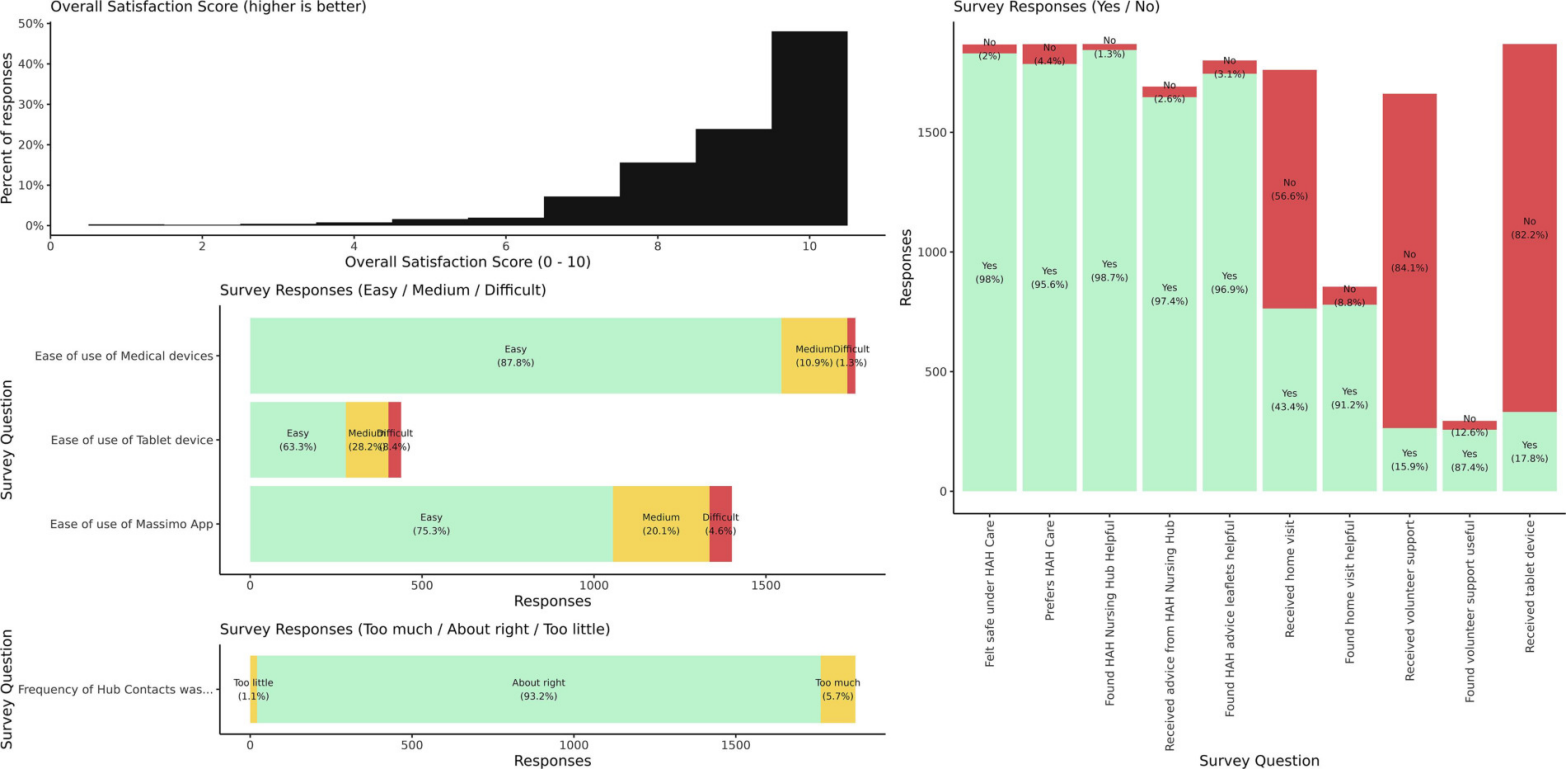
Patient experience survey responses. Most patients reported high satisfaction with HAH services (median 9, mean 8.95), felt safe under HAH care (98%), and preferred it over inpatient management (96%). Most responding patients found supplied medical devices and the Masimo App platform easy to use (87% and 75%, respectively), and most (93%) were satisfied with the frequency of contacts. The patient experience instrument is included as Supplementary Data Sheet 1.

A patient experience survey with 1,865 responses (62.8% response rate, 1,865/2,972) received following discharge showed that 95.8% of respondents reported a preference for HAH over inpatient care, while 98.3% felt safe under HAH care (see Table 6). Furthermore, 98.7% found the support of the HAH remote nursing hub helpful, and 92.2% felt that face-to-face nursing input was useful. A majority (93.2%) felt that the level of contact provided by the nursing hub was almost right, with 5.7% feeling that it was too frequent, and only 1.1% feeling that contacts were too infrequent. The median overall patient satisfaction rating (out of 10) over the study period was 9.0 (*n* = 1,381, IQR 8–10, mean 8.95). The survey responses are illustrated by Table 6 and Figures 5, 6.

**Table 6:** Quality-of-life survey.

	All responses			Responses at all timepoints		
Measure	Baseline (*n* = 2,411)	Discharge (*n* = 1,805)	1 Month (*n* = 1,656)	Baseline (*n* = 1,516)	Discharge (*n* = 1,516)	1 Month (*n* = 1,516)
Mobility						
No response	0 (0%)	0 (0%)	2 (0.1%)	0 (0%)	0 (0%)	1 (<0.1%)
No problem	1,366 (57%)	1,010 (56%)	932 (56%)	815 (54%)	838 (55%)	848 (56%)
Some problem	1,015 (42%)	779 (43%)	705 (43%)	686 (45%)	666 (44%)	652 (43%)
Severe problem	30 (1.2%)	16 (0.9%)	17 (1.0%)	15 (1.0%)	12 (0.8%)	15 (1.0%)
Self-care						
No response	0 (0%)	0 (0%)	1 (<0.1%)	0 (0%)	0 (0%)	0 (0%)
No problem	1,787 (74%)	1,383 (77%)	1,278 (77%)	1,136 (75%)	1,167 (77%)	1,175 (78%)
Some problem	554 (23%)	375 (21%)	337 (20%)	339 (22%)	309 (20%)	303 (20%)
Severe problem	70 (2.9%)	47 (2.6%)	40 (2.4%)	41 (2.7%)	40 (2.6%)	38 (2.5%)
Usual activity						
No response	2 (<0.1%)	1 (<0.1%)	1 (<0.1%)	1 (<0.1%)	1 (<0.1%)	0 (0%)
No problem	908 (38%)	812 (45%)	787 (48%)	608 (40%)	704 (46%)	734 (48%)
Some problem	1,300 (54%)	868 (48%)	757 (46%)	788 (52%)	709 (47%)	684 (45%)
Severe problem	201 (8.3%)	124 (6.9%)	111 (6.7%)	119 (7.8%)	102 (6.7%)	98 (6.5%)
Pain and discomfort						
No response	2 (<0.1%)	2 (0.1%)	1 (<0.1%)	2 (0.1%)	1 (<0.1%)	0 (0%)
No problem	1,631 (68%)	1,290 (71%)	1,167 (70%)	997 (66%)	1,076 (71%)	1,073 (71%)
Some problem	732 (30%)	480 (27%)	461 (28%)	477 (31%)	411 (27%)	418 (28%)
Severe problem	46 (1.9%)	33 (1.8%)	27 (1.6%)	40 (2.6%)	28 (1.8%)	25 (1.6%)
Anxiety/depression						
No response	4 (0.2%)	2 (0.1%)	2 (0.1%)	1 (<0.1%)	1 (<0.1%)	1 (<0.1%)
No problem	1,757 (73%)	1,396 (77%)	1,268 (77%)	1,084 (72%)	1,164 (77%)	1,168 (77%)
Some problem	620 (26%)	384 (21%)	372 (22%)	411 (27%)	332 (22%)	333 (22%)
Severe problem	30 (1.2%)	23 (1.3%)	14 (0.8%)	20 (1.3%)	19 (1.3%)	14 (0.9%)
Sleep quality						
No response	27 (1.1%)	46 (2.5%)	49 (3.0%)	18 (1.2%)	15 (1.0%)	21 (1.4%)
No problem	606 (25%)	463 (26%)	360 (22%)	353 (23%)	397 (26%)	338 (22%)
Some problem	1,603 (66%)	1,194 (66%)	1,171 (71%)	1,008 (66%)	1,008 (66%)	1,085 (72%)
Severe problem	175 (7.3%)	102 (5.7%)	76 (4.6%)	137 (9.0%)	96 (6.3%)	72 (4.7%)
Nocturnal waking						
Mean	2.18	1.86	1.73	2.16	1.83	1.74
Median	2	2	2	2	2	2
IQR	1–3	1–2	1–2	1–3	1–2	1–2
Count	2,048	1,530	1,440	1,354	1,330	1,349
Overall health perception						
Mean	61.5	70.5	70.7	61.9	70.7	71.0
Median	60	70	70	60	70	70
IQR	50–70	60–80	60–80	50–75	60–80	60–80
Count	2,401	1,799	1,650	1,515	1,510	1,511

Responses to a quality-of-life survey administered to HAH patients at onboarding, HAH discharge, and at 1 month following discharge. The first division includes any response with at least one completed question, while the second only includes responses from patients who provided responses with at least one completed question at every timepoint. Those who completed all three surveys provided broadly similar results to those who provided only partial responses.

**Table 7:** Patient experience survey.

Measure	No (%)		Yes (%)		Total (response rate)
Felt safe under HAH Care	23 (1.7%)		1,352 (98%)		1,375 (47%)
Prefers HAH Care	58 (4.2%)		1,318 (96%)		1,376 (47%)
Found HAH advice leaflets helpful	30 (2.2%)		1,304 (98%)		1,334 (46%)
Received advice from the HAH Nursing Hub	30 (2.4%)		1,218 (98%)		1,248 (43%)
Found the HAH Nursing Hub helpful	18 (1.3%)		1,359 (99%)		1,377 (47%)
Received home visit	743 (58%)		549 (42%)		1,292 (47%)
Found home visit helpful	47 (7.8%)		556 (92%)		603 (110%)
Received volunteer support	1,028 (84%)		202 (16%)		1,230 (42%)
Found volunteer support useful	29 (13%)		200 (87%)		229 (113%)
Received tablet device	1,123 (82%)		254 (18%)		1,377 (47%)
Measure	Easy	Medium	Difficult	Total (response rate)	
Ease of use of the Masimo App	769 (75%)	211 (20%)	50 (4.9%)		1,030 (35%)
Ease of use of Medical devices	1,143 (87%)	145 (11%)	22 (1.7%)		1,310 (45%)
Ease of use of the Tablet device	207 (63%)	90 (27%)	33 (10%)		330 (130%)
Measure	Not Enough	About Right	Too Much	Total (Response Rate)	
Frequency of Hub Contacts was..…	17 (1.2%)	1,282 (93%)	78 (5.7%)		1,377 (47%)
Characteristic	Mean	Median (IQR)	Range	Responses (%)	
Overall score	8.95	9 (8—10)	1–10		1, 381 (47%)

Responses were almost unanimous to questions on the safety, helpfulness, and preferability of HAH care. Some patients reported difficulty using the equipment and digital health platform. The mean overall experience score was 8.95. The results were limited by a response rate of approximately 47%, although some sub-questions were answered by patients who did not provide responses to the relevant subgrouping questions.

A majority of patients found the use of medical devices for home monitoring easy (87%), with only 1.7% reporting difficulty in using them. The Masimo Safetynet App and Platform were also reported as easy to use by 75% of patients, with only a small number of respondents finding it difficult to use (4.8%). Tablet devices were reported as easy to use by 63.2% of patients, with 28.2% reporting medium difficulty, and 8.4% finding them difficult to use.

Serial Eq-5D-3L quality-of-life questionnaires offered at HAH admission, discharge, and 1 month after discharge found significant differences in QoL outcomes over time for all measures tested, including mobility, ability to self-care, ability to carry out usual activities, pain and discomfort, anxiety and depression, sleep quality, nocturnal waking frequency, and overall health perception (see Table 7).

A *post hoc* all-pairs testing identified significant improvements in all outcomes, except mobility between admission and discharge, and in nocturnal waking frequency and overall health perception between discharge and 1-month follow-up. In addition, mobility, self-care, ability to carry out usual activities, pain and discomfort, anxiety and depression, and nocturnal waking frequency were significantly better at the 1-month follow-up compared with admission.

Overall QoL scores improved from 61.5 at baseline to 70.5 at offboarding (*p* < 6 × 10^−7^). This improvement was maintained at 1 month, at 70.7 (*p* ns). Notably, the response rate of the QoL questionnaire declined over time, with 79.3% completions at admission (2,357/2,972), 58.3% at discharge (1,732/2,972), and 53.6% (1,593/2,972) at 1-month follow-up; 49.3% (1,466/2,972) of respondents completed all surveys.

### Safety outcomes

The rates of escalation back to IP while under HAH care were low at 14% in both AD (55/398; 95% CI 11%–18%) and ESD (157/1,090; 95% CI 12%–17%) groups; many of these escalations were triggered from within the HAH service often related to symptoms or remote monitoring. Safety outcomes are presented in the lower half of Table 5.

All-cause 30-day readmission rates tended to be significantly lower in HAH cohorts than in matched controls (AD: OR 0.41, 95% CI 0.24–0.68, *p* < 5 × 10^−4^; ESD: OR 0.62, 95% CI 0.46–0.84, *p* < 2 × 10^−3^. The pooled HAH cohort was also at significantly lesser risk of readmission compared with the pseudo-experimental IP cohort: OR 0.19 (8.1% vs. 31.8%, 95% CI 0.11-0.32, *p* < 2 × 10^−9^, *n* = 1,577, of which 85 were decliners).

Despite escalations, the total time in hospital from any cause over the 90 days from initial presentation was also significantly shorter for HAH patients (AD: 4.08 days fewer, 95% CI 2.81–5.36, *p* < 6 × 10^−10^; ESD: 2.29 days fewer, 95% CI 1.37–3.21, *p* < 2 × 10^−6^), and HAH patients also had fewer calendar days with any hospital contact (AD: 3.97 days fewer, 95% CI 2.64–5.30, *p* < 8 × 10^−9^; ESD: 2.20 days fewer, 95% CI 1.25–3.16, *p* < 7 × 10^−6^).

There was no evidence of increased risk of death in the HAH cohort; in fact, mortality was lower in the HAH cohorts—the odds ratio for 90-day mortality in AD was 0.34 (95% CI 0.20–0.55, *p* < 6 × 10^−6^), and in ESD, it was 0.45 (95% CI 0.35–0.56, *p* < 7 × 10^−11^). Comparisons with the pseudo-experimental IP cohort also tended toward lower mortality, but it did not reach significance (OR 0.69, 95% CI 0.39–1.28, *p* = 0.21, *n* = 1,577 of which 85 were decliners).

Pooled statistics across AD and ESD were in line with pathway-specific statistics: the overall odds ratio for 30-day readmissions of any cause was 0.55 (95% CI 0.42–0.70, *p* < 3 × 10^−6^), while time in hospital over 90 days was 2.64 days fewer (95% CI 1.87–3.40, *p* < 2 × 10^−11^), with 2.54 fewer calendar days (95% CI 1.75–3.33, *p* < 4 × 10^−10^). The overall odds ratio for mortality from any cause was 0.43 (95% CI 0.35–0.53, *p* < 3 × 10^−16^).

### Real-world impact on inpatient service structure

**Figure 7:**
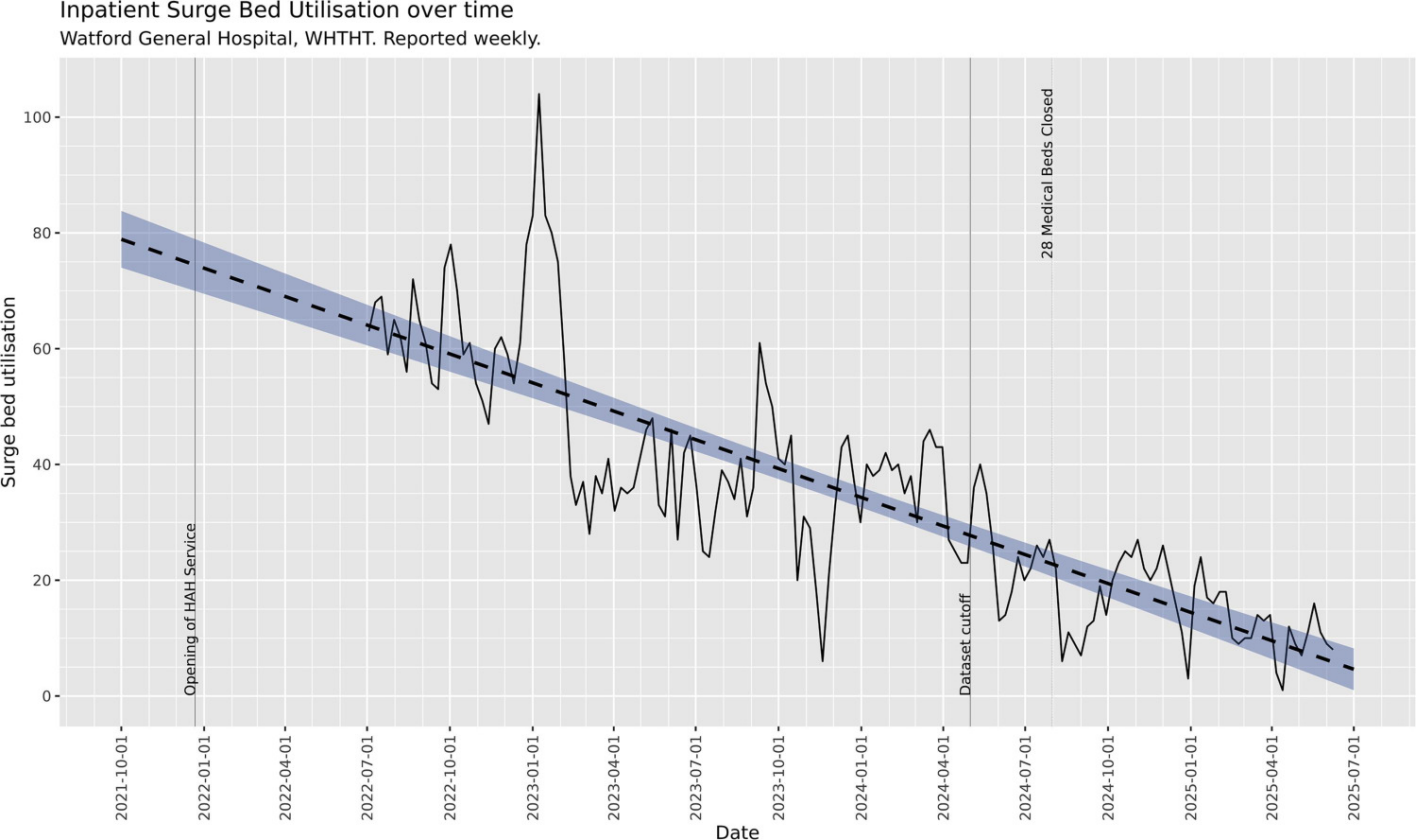
Inpatient surge bed utilisation over time. Following implementation of the HAH Program, the utilisation of surge beds—emergency additional hospital capacity created in response to exceptional demand—has trended downwards. This trend persisted even after the closure of a 28-bedded unit, representing approximately 5% of inpatient capacity.

Over the study period, as part of a package of changes including development and expansion of HAH services, improved integration of HAH pathways into medical patient flow, and other improvements related to flow throughout the hospital, measurable performance improvements have been achieved, and the WHTHT has been able to reduce mean surge bed occupancy from 65 to 20 between July 2022 and August 2025. This has meant that shortly after the conclusion of this study, the hospital was able to permanently close one 28-bedded general medical inpatient ward and redeploy resources to other areas with no increase in surge capacity utilisation, as shown in Figure 7. In other words, HAH has allowed our hospital to simultaneously reduce inpatient bedded capacity and improve performance partly because of the HAH intervention.

## Discussion

The current study is one of the largest analyses of a virtual hospital or hospital at home model of care, with nearly 3,000 patients involved in a real-world analysis. The key findings are that HAH care shortened the mean inpatient length of stay in an early supported discharge model from 9.3 to 6.2 days, a reduction of 3.1 days (33%), with much larger proportional length of stay reductions in admission avoidance and admission prevention models. This was highly cost-effective with a significant impact on the overall clinical performance of the hospital. This model of care was safe and highly acceptable to patients with improvement in repeated quality-of-life metrics following onboarding. In particular, the patient experience data are noteworthy for the very high level of patient satisfaction, allowing for the caveat that comparable data were not available for control patients.

There was no safety signal in terms of mortality. In fact, intriguingly, mortality was significantly lower in the HAH cohort. While this may be partially related to imperfect matching, given the evidence of good matching quality and broad consistency between the matched and parallel pseudo-experimental group, it may also indicate that HAH is safer as patients avoid well-documented harms related to hospital admission, including hospital-associated infection, delirium, and deconditioning (18). Indeed, while evidence is tentative, subject to bias, and drawn from secondary or safety outcomes, several other studies have found lower mortality in HAH cohorts (19–21).

Despite earlier discharge, all-cause 30-day readmissions were also lower following HAH intervention compared with direct discharge from inpatient care. There were also escalations back to inpatient care from within HAH: many of these were triggered from within the HAH service, for example after a home visit, patient-reported concerns, or issues related to remote monitoring data, and these are important to maintain safety and are also likely a reflection of acuity mix. Comparable values are scarcely reported, but one study that defined “re-presentation” as a return to BAM hospital within 2 days of discharge reported a lower rate of 7.9% (20).

A detailed analysis shows that HAH patients were well matched to control patients, and the doubly robust design allows for the correction of residual imbalances to improve accuracy and confidence in these results. Compellingly, a separate pseudo-experimental comparison against a group of patients who refused offers of HAH care for non-medical reasons provides results across the main outcome measures in close agreement, lending credibility to the regression-adjusted propensity score matched findings.

The real-world cost per bed day was £119, which compared very favourably with the cost of an inpatient bed day (£569). The overall cost per episode of care also showed a significant saving, and this was particularly pronounced in the prevention of admission virtual ward with a saving of £3.79 m over the 33 months of the analysis. This contrasts with a previous study from Wrightington, Wigan, and Leigh (WWL) Teaching Hospitals, which reported a cost per bed day in the Virtual Ward of £935, nearly eight times more (6). As we pointed out at the time, the WWL economic analysis failed to capture the potential of a mature HAH service because of its low occupancy rate of only 25% design capacity and because of the likelihood that the analysis was performed during the virtual ward learning curve (22). If our finding were replicated across the NHS in England and the United Kingdom, the savings to the system would be hundreds of thousands of bed days every year, representing hundreds of millions of pounds.

Several previous studies from the United Kingdom provide useful context to these results. A previous randomised controlled trial (RCT) of HAH in 1,050 older adults in England delivered a Comprehensive Geriatric Assessment (CGA) at home as an alternative to hospital admission and showed safety, efficacy, and cost-effectiveness, with lower rates of residential care and delirium in the HAH population (23). An evaluation of HAH care in the South East England region showed an overall positive impact on the healthcare system but did not include the level of matched individual patient level data that we provide (24). An analysis of three geriatric HAH services in Scotland prior to the COVID-19 pandemic making use of a propensity-score matching (PSM) and principally routinely collected data (RCD) found mixed but generally greater 6-month overall healthcare expenditure associated with HAH care, and possibly higher mortality, subject to the caveat that unmeasured confounders may have biased mortality findings (25). These studies are more directly comparable due to their setting in the British healthcare system, and while they provide some reassurance, there are limited data on resource utilisation or for high-acuity, broad applicability services such as those presented here.

International comparisons are limited, with significant variation in findings that may reflect the wide range of models utilised and patient populations treated. A 91-patient RCT in the US of an HAH service showed reduced cost, healthcare use, and readmissions while increasing physical activity compared with usual hospital care (26). A large Australian regional study including 2,095 HAH episodes in South Australia used a similar approach in applying mainly RCD to an inverse probability weighting (IPW) matching design and found lower 28-day readmission and possibly 30-day mortality rates, as well as providing valuable qualitative findings on patient experience (20). A large Spanish regional study that included 31,901 HAH episodes in Catalonia prior to the global COVID-19 pandemic similarly made use of RCD and a PSM design and found an increased risk of 30-day readmission and greater total healthcare expenditure but a lower risk of 30-day mortality (19). Analyses of a single COVID-19 HAH service in Queensland, Australia, estimated a $201 AUD saving per HAH admission (27). A 543 patient study in Canada evaluated patient and caregiver experience of a hospital at home model of care (28). While these services operated a range of models and in diverse settings, they have broadly confirmed the practicality and safety of the HAH concept, with several studies also suggesting potential mortality benefits, although none have specifically set out to investigate this as a primary outcome. While evidence on cost and resource utilisation is more mixed, studies demonstrating reduced costs have shown a similar magnitude of benefit.

### Limitations

Several potential limitations in this must be acknowledged. Model misspecification in propensity matching can introduce bias in the results. Comprehensive details are not available on specific disease biomarkers (such as spirometry data or BNP) or on functional markers of frailty (such as the degree of formal or informal care), creating the potential for unmeasured confounders to introduce bias. The assumption that matching criteria are sufficient to fully explain the likelihood of allocation to either control or treatment groups (e.g., conditional independence) is unlikely to conclusively provide a satisfactory answer either theoretically or numerically. In addition, if a large proportion of patients who are likely to be admitted to HAH have been identified and onboarded and are therefore not eligible for the control group, a lack of common support may limit comparability between groups. The choice of parameters and algorithm for match selection can also introduce both bias and model dependence on the selection of matching algorithms and parameters.

Although residual imbalance in measured confounders due to imperfect matching has been addressed by using the matched cohort as the basis for regression modelling—a doubly robust design—this will not be able to address unmeasured confounders. The use of regression modelling also introduces the possibility of bias due to model misspecification. While more complex or specific indicators of frailty and illness severity could have provided for finer grained matching and may have reduced the possibility of unmeasured confounding, the need to determine these parameters would have significantly restricted the achievable cohort and comparator group sizes compared with an approach that makes use of principally routinely collected data.

While efforts have been made to ensure that patient selection is consistent and based on clinically valid criteria, it is possible that patient selection could introduce bias. Patient selection also has a significant influence on generalisability, and while we have attempted to maximise the scope of eligibility through the design of our service and use of a high-acuity model, a careful assessment of suitability and care needs is essential, and HAH care is implicitly restricted to those who are willing and able to undertake the responsibilities it requires of them.

The quality of matching has been examined using both graphical (including balance and love plots) and numerical methods, which have shown close matching across all parameters. Concerns about the quality of matching and model validity are also partly mitigated by the doubly robust design, which is intended to reduce model dependence. Further reassurance is provided by the findings of the parallel pseudo-experimental cohort, which are in close agreement with the matched cohort for most measures, lending strength to the reliability of these results.

The pseudo-experimental cohort subject used to validate the principal results is limited by its small size, which restricts its statistical power, and by the risk of selection bias; it is possible that patients who refused HAH differed in significant ways from those who availed it. As no regression corrections are performed on this cohort, random imbalance in its demography may have a greater influence on the results than in a larger group.

This study was performed retrospectively in a single centre with a particular HAH care model. However, SWH is broadly typical of medium-sized NHS health systems in England, including a 521-bed district general hospital and an active community partner. It is notable that both our cohort and the population-weighted median IMD score for the sub-ICB area from which most of our patients were drawn are significantly less deprived than the median for England, at around the 66th–70th decile, and it is plausible that more deprived patients may find HAH care more challenging. While significant variation persists in the scope and acuity of HAH services nationally, we are hopeful that the broad range of indications managed under the combined pathways (ABC, ARI, and HF) contributes to evidence in favour of broad, high-acuity medical speciality-led HAH services.

Patient experience and acceptability surveys are subject to some limitations due to patient response rates; in particular, the serial-measures QoL survey is complicated by declining response rates over time—which fell to nearly 50% at 1 month post discharge. In addition, the service-specific use of these survey instruments over simpler tools used in routine service monitoring means that no equivalent data are available for any comparable inpatient cohort. It is possible that the likelihood of survey completion is confounded by factors such as frailty, functional impairment, and satisfaction. It is often anecdotally reported that dissatisfied respondents are more likely to complete surveys, and while results from partial and complete responses to the serial quality-of-life surveys were similar, it is impossible to determine what responses those who completed no surveys would have provided. Further analysis could assess and seek to correct for structural differences between responders and non-responders. Future work that relies on survey data can also explore the reasons for non-response, as well as exploring other methodologies such as more qualitative approaches. It is reassuring that the results are broadly unanimous in terms of support for HAH care, implying that even in the worst case where all non-respondents were dissatisfied, mean approval would remain positive.

## Conclusion

Overall, this study is the largest study of a Virtual Ward programme HAH service to date in England. It shows that a high-acuity HAH service significantly and meaningfully reduces the length of stay for suitable patients and delivers major cost savings in terms of overall care delivery both during the index admission and over the subsequent 90 days. It is safe and highly acceptable to patients and may also confer benefits in terms of reduced risk of readmission and possibly mortality. This service should be prioritised in ongoing NHS development plans. The data in this study provide strong support for a wider rollout of this model of care, both in the NHS and around the world. Further studies are required in other healthcare environments, and a larger, interventional randomised trial should be considered.

## Data Availability

The datasets used for this work are not publicly available due to medical confidentiality. Requests to access the datasets should be directed to the corresponding author.

## Footnotes

^1^See Supplementary Materials for ICD10 code list.

^2^See Supplemental Materials for survey instruments.

^3^See Supplemental Data, “Population-weighted mean IMD by sub-ICB area.”
