# Supplementary material for: Real-world outcomes from 2,905 episodes of hospital at home care: a propensity-matched cohort study
Source: Front Digit Health. 2026 Apr 8;8:1716319. doi: 10.3389/fdgth.2026.1716319 (PMC13101057; doi:10.3389/fdgth.2026.1716319)
Supplement: Supplementary file 1 [file Table1.docx]

TABLE 1 Inclusion and exclusion criteria.

| Pathway | Inclusion Criteria | Exclusion Criteria |
| --- | --- | --- |
| HF | Diagnosis of Heart Failure requiring fluid status optimisation | Diagnosis of Severe Aortic Stenosis |
|  | Diagnosis of Atrial Fibrillation, or atrial flutter, requiring rate or rhythm control optimisation |  |
| ABC | Diagnosis of Asthma, Bronchiectasis, or COPD | Diagnosis of ILD (initially) |
| ARI | Diagnosis of Pneumonia, Viral Pneumonitis, or COVID-19 | No specific criteria |
|  | Diagnosis of other medical problems deemed suitable for remote management |  |
| Overall | Pathway Specific Criteria | Inability to deliver required medical and care needs at home |
|  |  | Patient refusal |
|  |  | Inability to manage self-monitoring |
|  |  | Physical or mental difficulties impairing the ability to seek medical attention in case of deterioration |

Pathway-specific and whole-service inclusion and exclusion criteria during the study period.
