# Supplementary material for: Real-world outcomes from 2,905 episodes of hospital at home care: a propensity-matched cohort study
Source: Front Digit Health. 2026 Apr 8;8:1716319. doi: 10.3389/fdgth.2026.1716319 (PMC13101057; doi:10.3389/fdgth.2026.1716319)
Supplement: Supplementary file 2 [file Table2.docx]

## Supplemental 2: ICD10 codes used for selection of IP Controls

| **ICD10 Code** | **Diagnosis Text** |  | **ICD10 Code** | **Diagnosis Text** |  |
| --- | --- | --- | --- | --- | --- |
| **A310** | Pulmonary mycobacterial infection |  | **J150** | Pneumonia due to Klebsiella pneumoniae |  |
| **A481** | Legionnaires disease |  | **J151** | Pneumonia due to Pseudomonas |  |
| **E877** | Fluid overload |  | **J152** | Pneumonia due to staphylococcus |  |
| **I083** | Combined disorders of mitral, aortic and tricuspid valves |  | **J154** | Pneumonia due to other streptococci |  |
| **I110** | Hypertensive heart disease with (congestive) heart failure |  | **J156** | Pneumonia due to other Gram-negative bacteria |  |
| **I132** | Hypertensive heart and renal disease with both (congestive) heart failure and renal failure |  | **J157** | Pneumonia due to Mycoplasma pneumoniae |  |
| **I139** | Hypertensive heart and renal disease, unspecified |  | **J181** | Lobar pneumonia, unspecified |  |
| **I210** | Acute transmural myocardial infarction of anterior wall |  | **J189** | Pneumonia, unspecified |  |
| **I211** | Acute transmural myocardial infarction of inferior wall |  | **J22X** | Unspecified acute lower respiratory infection |  |
| **I214** | Acute subendocardial myocardial infarction |  | **J40X** | Bronchitis, not specified as acute or chronic |  |
| **I219** | Acute myocardial infarction, unspecified |  | **J438** | Other emphysema |  |
| **I249** | Acute ischaemic heart disease, unspecified |  | **J439** | Emphysema, unspecified |  |
| **I251** | Atherosclerotic heart disease |  | **J440** | Chronic obstructive pulmonary disease with acute lower respiratory infection |  |
| **I255** | Ischaemic cardiomyopathy |  | **J441** | Chronic obstructive pulmonary disease with acute exacerbation, unspecified |  |
| **I269** | Pulmonary embolism without mention of acute cor pulmonale |  | **J448** | Other specified chronic obstructive pulmonary disease |  |
| **I420** | Dilated cardiomyopathy |  | **J449** | Chronic obstructive pulmonary disease, unspecified |  |
| **I428** | Other cardiomyopathies |  | **J450** | Predominantly allergic asthma |  |
| **I438** | Cardiomyopathy in other diseases classified elsewhere |  | **J459** | Asthma, unspecified |  |
| **I447** | Left bundle-branch block, unspecified |  | **J46X** | Status asthmaticus |  |
| **I453** | Trifascicular block |  | **J47X** | Bronchiectasis |  |
| **I471** | Supraventricular tachycardia |  | **J690** | Pneumonitis due to food and vomit |  |
| **I480** | Paroxysmal atrial fibrillation |  | **J704** | Drug-induced interstitial lung disorders, unspecified |  |
| **I481** | Persistent atrial fibrillation |  | **J841** | Other interstitial pulmonary diseases with fibrosis |  |
| **I489** | Atrial fibrillation and atrial flutter, unspecified |  | **J90X** | Pleural effusion, not elsewhere classified |  |
| **I500** | Congestive heart failure |  | **J9600** | Acute respiratory failure; Type I [hypoxic] |  |
| **I501** | Left ventricular failure |  | **J9611** | Chronic respiratory failure; Type II [hypercapnic] |  |
| **I509** | Heart failure, unspecified |  | **J9691** | Respiratory failure, unspecified; Type II [hypercapnic] |  |
| **I518** | Other ill-defined heart diseases |  | **R002** | Palpitations |  |
| **J069** | Acute upper respiratory infection, unspecified |  | **R011** | Cardiac murmur, unspecified |  |
| **J100** | Influenza with pneumonia, seasonal influenza virus identified |  | **R042** | Haemoptysis |  |
| **J101** | Influenza with other respiratory manifestations, seasonal influenza virus identified |  | **R05X** | Cough |  |
| **J108** | Influenza with other manifestations, seasonal influenza virus identified |  | **R060** | Dyspnoea |  |
| **J121** | Respiratory syncytial virus pneumonia |  | **R570** | Cardiogenic shock |  |
| **J122** | Parainfluenza virus pneumonia |  | **T820** | Mechanical complication of heart valve prosthesis |  |
| **J13X** | Pneumonia due to Streptococcus pneumoniae |  | **U071** | Emergency use of U07.1 |  |
| **J14X** | Pneumonia due to Haemophilus influenzae |  |  |  |  |
