# Supplementary material for: Real-world outcomes from 2,905 episodes of hospital at home care: a propensity-matched cohort study
Source: Front Digit Health. 2026 Apr 8;8:1716319. doi: 10.3389/fdgth.2026.1716319 (PMC13101057; doi:10.3389/fdgth.2026.1716319)
Supplement: Supplementary file 3 [file Table3.docx]

## Supplemental 3: ICD10 codes not included for selection of IP Controls

| **ICD10 Code** | **Diagnosis Text** | **Reasoning** |
| --- | --- | --- |
| **N179** | Acute renal failure, unspecified | Outside HAH Criteria |
| **I10X** | Essential (primary) hypertension | Outside HAH Criteria |
| **N390** | Urinary tract infection, site not specified | Outside HAH Criteria; Low case number (<1%) |
| **A099** | Gastroenteritis and colitis of unspecified origin | Outside HAH Criteria; Low case number (<0.5%) |
| **A419** | Sepsis, unspecified | Outside HAH Criteria; Low case number (<0.5%) |
| **E871** | Hypo-osmolality and hyponatraemia | Outside HAH Criteria; Low case number (<0.5%) |
| **R55X** | Syncope and collapse | Outside HAH Criteria; Low case number (<0.5%) |
| **I209** | Angina pectoris, unspecified | Low case number (<0.5%) |
| **R296** | Tendency to fall, not elsewhere classified | Outside HAH Criteria; Low case number (<0.5%) |
| **E876** | Hypokalaemia | Outside HAH Criteria; Low case number (<0.5%) |
| **F419** | Anxiety disorder, unspecified | Outside HAH Criteria; Low case number (<0.5%) |
| **R11X** | Nausea and vomiting | Outside HAH Criteria; Low case number (<0.5%) |
| **I460** | Cardiac arrest with successful resuscitation | Outside HAH Criteria; Low case number (<0.5%) |
| **E115** | Type 2 diabetes mellitus; With peripheral circulatory complications | Outside HAH Criteria; Low case number (~0.1%) |
| **G409** | Epilepsy, unspecified | Outside HAH Criteria; Low case number (~0.1%) |
| **H431** | Vitreous haemorrhage | Outside HAH Criteria; Low case number (~0.1%) |
| **M7986** | Other specified soft tissue disorders; Lower leg | Outside HAH Criteria; Low case number (~0.1%) |
| **R101** | Pain localized to upper abdomen | Outside HAH Criteria; Low case number (~0.1%) |
| **T840** | Mechanical complication of internal joint prosthesis | Outside HAH Criteria; Low case number (~0.1%) |
| **A415** | Sepsis due to other Gram-negative organisms | Outside HAH Criteria; Low case number (single case) |
| **A418** | Other specified sepsis | Outside HAH Criteria; Low case number (single case) |
| **B279** | Infectious mononucleosis, unspecified | Outside HAH Criteria; Low case number (single case) |
| **B349** | Viral infection, unspecified | Outside HAH Criteria; Low case number (single case) |
| **C349** | Malignant neoplasm: Bronchus or lung, unspecified | Outside HAH Criteria; Low case number (single case) |
| **C787** | Secondary malignant neoplasm of liver and intrahepatic bile duct | Outside HAH Criteria; Low case number (single case) |
| **D500** | Iron deficiency anaemia secondary to blood loss (chronic) | Outside HAH Criteria; Low case number (single case) |
| **D693** | Idiopathic thrombocytopenic purpura | Outside HAH Criteria; Low case number (single case) |
| **E109** | Type 1 diabetes mellitus; Without complications | Outside HAH Criteria; Low case number (single case) |
| **G403** | Generalized idiopathic epilepsy and epileptic syndromes | Outside HAH Criteria; Low case number (single case) |
| **I802** | Phlebitis and thrombophlebitis of other deep vessels of lower extremities | Outside HAH Criteria; Low case number (single case) |
| **K720** | Acute and subacute hepatic failure | Outside HAH Criteria; Low case number (single case) |
| **K852** | Alcohol-induced acute pancreatitis | Outside HAH Criteria; Low case number (single case) |
| **M316** | Other giant cell arteritis | Outside HAH Criteria; Low case number (single case) |
| **M5436** | Sciatica; Lumbar region | Outside HAH Criteria; Low case number (single case) |
| **M7966** | Pain in limb; Lower leg | Outside HAH Criteria; Low case number (single case) |
| **M7980** | Other specified soft tissue disorders; Multiple sites | Outside HAH Criteria; Low case number (single case) |
| **M7983** | Other specified soft tissue disorders; Forearm | Outside HAH Criteria; Low case number (single case) |
| **R092** | Respiratory arrest | Outside HAH Criteria; Low case number (single case) |
| **R630** | Anorexia | Outside HAH Criteria; Low case number (single case) |
| **R935** | Abnormal findings on diagnostic imaging of other abdominal regions, including retroperitoneum | Outside HAH Criteria; Low case number (single case) |
| **S0660** | Traumatic subarachnoid haemorrhage; without open intracranial wound | Outside HAH Criteria; Low case number (single case) |
| **S4220** | Fracture of upper end of humerus; closed | Outside HAH Criteria; Low case number (single case) |
| **Z432** | Attention to ileostomy | Outside HAH Criteria; Low case number (single case) |
