# Supplementary material for: Real-world outcomes from 2,905 episodes of hospital at home care: a propensity-matched cohort study
Source: Front Digit Health. 2026 Apr 8;8:1716319. doi: 10.3389/fdgth.2026.1716319 (PMC13101057; doi:10.3389/fdgth.2026.1716319)
Supplement: Supplementary file 5 [file Datasheet1.pdf]

Virtual Hospital – Patient Experience Questionnaire

HF/COPD/ARI – which patient group?

Overall

On a scale of **1-10** where 1 is a very poor experience and 10 is a very good experience how would you describe your experience of the virtual hospital?

Clinical

1. The aim of virtual hospital is to get patients out of hospital earlier while giving them safe care in their home. Do you feel going home sooner from hospital helped you? **Yes/No**
2. Did you feel safe as a patient in our Virtual hospital? **Yes/No**

Contact/communication

1. Thinking of your contact with the hub team, did you find that contact to be helpful? **Yes/No**
2. In terms of your communication with the hub team do you think the frequency of phone calls and interactions was: **too much / about right / too little**?
3. When you needed help or advice did you get the answers you needed? **yes/no**
4. Did you find the patient information leaflet helpful? **Yes / no** Would you like to suggest any changes?
5. Did you receive a visit from one of our community teams? **Yes/no**
6. If so which: COPD, HF, phlebotomy, rapid response, district nurse, other?
7. Did you find the community team helpful? **Yes/no**
8. Did you receive a call or visit from one of our partners in the voluntary section? **Yes/no**
9. If so which:
10. Did you find the voluntary sector input helpful? **Yes/no**

IT/Equipment

1. How easy was the Massimo App to use? **Easy / Medium / Difficult**
2. Did you have a VH tablet on loan? **Yes/no**
3. How easy was this to use? **Easy / Medium / Difficult**
4. How easy or difficult were the clinical devices to use? **Easy / Medium / Difficult**

Do you have any other points you would like to raise about equipment or IT?

PLEASE TURN OVER

Is there anything in particular that you feel worked very well?

What do you feel we could do better?
