# Supplementary material for: Real-world outcomes from 2,905 episodes of hospital at home care: a propensity-matched cohort study
Source: Front Digit Health. 2026 Apr 8;8:1716319. doi: 10.3389/fdgth.2026.1716319 (PMC13101057; doi:10.3389/fdgth.2026.1716319)
Supplement: Supplementary file 6 [file Datasheet2.docx]

## Supplemental Material: List of R packages used for analysis

### Core

- R Core Team (2024). *R: A Language and Environment for Statistical Computing*. R Foundation for Statistical Computing, Vienna, Austria. [https://www.R-project.org/](https://www.r-project.org/).

### Development

- Henry L, Wickham H (2024). *rlang: Functions for Base Types and Core R and 'Tidyverse' Features*. R package version 1.1.4, <https://github.com/r-lib/rlang>, [https://rlang.r-lib.org](https://rlang.r-lib.org/).
- Wickham H (2023). *conflicted: An Alternative Conflict Resolution Strategy*. R package version 1.2.0, <https://github.com/r-lib/conflicted>, <https://conflicted.r-lib.org/>.
- Wickham H, Hester J, Chang W, Bryan J (2022). *devtools: Tools to Make Developing R Packages Easier*. R package version 2.4.5, <https://github.com/r-lib/devtools>, <https://devtools.r-lib.org/>.
- Wickham H, Bryan J, Barrett M, Teucher A (2024). *usethis: Automate Package and Project Setup*. R package version 3.1.0, <https://github.com/r-lib/usethis>, [https://usethis.r-lib.org](https://usethis.r-lib.org/).

### Parallelization

- Bengtsson H (2021). “A Unifying Framework for Parallel and Distributed Processing in R using Futures.” *The R Journal*, *13*(2), 208-227. doi:10.32614/RJ-2021-048 <https://doi.org/10.32614/RJ-2021-048>, <https://doi.org/10.32614/RJ-2021-048>.
- Wickham H, Henry L (2023). *purrr: Functional Programming Tools*. R package version 1.0.2, <https://github.com/tidyverse/purrr>, <https://purrr.tidyverse.org/>.
- Vaughan D, Dancho M (2022). *furrr: Apply Mapping Functions in Parallel using Futures*. R package version 0.3.1, <https://furrr.futureverse.org/>, <https://github.com/DavisVaughan/furrr>.

### Utility

- Wickham H, Averick M, Bryan J, Chang W, McGowan LD, François R, Grolemund G, Hayes A, Henry L, Hester J, Kuhn M, Pedersen TL, Miller E, Bache SM, Müller K, Ooms J, Robinson D, Seidel DP, Spinu V, Takahashi K, Vaughan D, Wilke C, Woo K, Yutani H (2019). “Welcome to the tidyverse.” *Journal of Open Source Software*, *4*(43), 1686. doi:10.21105/joss.01686 <https://doi.org/10.21105/joss.01686>.
- Wickham H (2023). *forcats: Tools for Working with Categorical Variables (Factors)*. R package version 1.0.0, <https://github.com/tidyverse/forcats>, <https://forcats.tidyverse.org/>.
- Wickham H (2023). *stringr: Simple, Consistent Wrappers for Common String Operations*. R package version 1.5.1, <https://github.com/tidyverse/stringr>, [https://stringr.tidyverse.org](https://stringr.tidyverse.org/).
- Wickham H, François R, Henry L, Müller K, Vaughan D (2023). *dplyr: A Grammar of Data Manipulation*. R package version 1.1.4, <https://github.com/tidyverse/dplyr>, [https://dplyr.tidyverse.org](https://dplyr.tidyverse.org/).
- Wickham H, Vaughan D, Girlich M (2024). *tidyr: Tidy Messy Data*. R package version 1.3.1, <https://github.com/tidyverse/tidyr>, [https://tidyr.tidyverse.org](https://tidyr.tidyverse.org/).
- Müller K, Wickham H (2023). *tibble: Simple Data Frames*. R package version 3.2.1, <https://github.com/tidyverse/tibble>, <https://tibble.tidyverse.org/>.
- Grolemund G, Wickham H (2011). “Dates and Times Made Easy with lubridate.” *Journal of Statistical Software*, *40*(3), 1-25. <https://www.jstatsoft.org/v40/i03/>.
- Eddelbuettel D (2024). *digest: Create Compact Hash Digests of R Objects*. R package version 0.6.37, <https://dirk.eddelbuettel.com/code/digest.html>, <https://github.com/eddelbuettel/digest>.

### IO

- Wickham H, Bryan J (2023). *readxl: Read Excel Files*. R package version 1.4.3, <https://github.com/tidyverse/readxl>, [https://readxl.tidyverse.org](https://readxl.tidyverse.org/).
- Ooms J (2024). *writexl: Export Data Frames to Excel 'xlsx' Format*. R package version 1.5.1, [https://ropensci.r-universe.dev/writexlhttps://docs.ropensci.org/writexl/](https://ropensci.r-universe.dev/writexlhttps:/docs.ropensci.org/writexl/).
- Müller K (2020). *here: A Simpler Way to Find Your Files*. R package version 1.0.1, <https://github.com/r-lib/here>, <https://here.r-lib.org/>.
- Wickham H, Hester J, Bryan J (2024). *readr: Read Rectangular Text Data*. R package version 2.1.5, <https://github.com/tidyverse/readr>, [https://readr.tidyverse.org](https://readr.tidyverse.org/).

### Plotting

- Wickham H (2016). *ggplot2: Elegant Graphics for Data Analysis*. Springer-Verlag New York. ISBN 978-3-319-24277-4, [https://ggplot2.tidyverse.org](https://ggplot2.tidyverse.org/).
- Kassambara A (2023). *ggpubr: 'ggplot2' Based Publication Ready Plots*. R package version 0.6.0, <https://rpkgs.datanovia.com/ggpubr/>.
- Slowikowski K (2024). *ggrepel: Automatically Position Non-Overlapping Text Labels with 'ggplot2'*. R package version 0.9.6, <https://github.com/slowkow/ggrepel>, <https://ggrepel.slowkow.com/>.
- Dayim A (2024). *consort: Create Consort Diagram*. R package version 1.2.2, <https://github.com/adayim/consort/>.
- Pedersen T (2024). *patchwork: The Composer of Plots*. R package version 1.3.0, <https://github.com/thomasp85/patchwork>, [https://patchwork.data-imaginist.com](https://patchwork.data-imaginist.com/).

### Tables

- Sjoberg D, Whiting K, Curry M, Lavery J, Larmarange J (2021). “Reproducible Summary Tables with the gtsummary Package.” *The R Journal*, *13*, 570-580. doi:10.32614/RJ-2021-053 <https://doi.org/10.32614/RJ-2021-053>, <https://doi.org/10.32614/RJ-2021-053>.
- Iannone R, Cheng J, Schloerke B, Hughes E, Lauer A, Seo J, Brevoort K, Roy O (2024). *gt: Easily Create Presentation-Ready Display Tables*. R package version 0.11.1, <https://github.com/rstudio/gt>, [https://gt.rstudio.com](https://gt.rstudio.com/).

### Geospatial

- Giraud T (2022). “osrm: Interface Between R and the OpenStreetMap-Based Routing Service OSRM.” *Journal of Open Source Software*, *7*(78), 4574. doi:10.21105/joss.04574 <https://doi.org/10.21105/joss.04574>, <https://doi.org/10.21105/joss.04574>.
- Dunnington D (2023). *ggspatial: Spatial Data Framework for ggplot2*. R package version 1.1.9, <https://github.com/paleolimbot/ggspatial>, <https://paleolimbot.github.io/ggspatial/>.
- Pebesma E, Bivand R (2023). *Spatial Data Science: With applications in R*. Chapman and Hall/CRC. doi:10.1201/9780429459016 <https://doi.org/10.1201/9780429459016>, <https://r-spatial.org/book/>. Pebesma E (2018). “Simple Features for R: Standardized Support for Spatial Vector Data.” *The R Journal*, *10*(1), 439-446. doi:10.32614/RJ-2018-009 <https://doi.org/10.32614/RJ-2018-009>, <https://doi.org/10.32614/RJ-2018-009>.
- Tennekes M (2018). “tmap: Thematic Maps in R.” *Journal of Statistical Software*, *84*(6), 1-39. doi:10.18637/jss.v084.i06 <https://doi.org/10.18637/jss.v084.i06>.
- Tennekes M (2021). *tmaptools: Thematic Map Tools*. R package version 3.1-1, <https://github.com/mtennekes/tmaptools>.

### Regression

- Heinze G, Ploner M, Jiricka L, Steiner G (2025). *logistf: Firth's Bias-Reduced Logistic Regression*. R package version 1.26.1, <https://github.com/georgheinze/logistf>.

### Bootstrapping

- Angelo Canty, B. D. Ripley (2024). *boot: Bootstrap R (S-Plus) Functions*. R package version 1.3-31. A. C. Davison, D. V. Hinkley (1997). *Bootstrap Methods and Their Applications*. Cambridge University Press, Cambridge. ISBN 0-521-57391-2, <doi:10.1017/CBO9780511802843>.
- Peng RD (2024). *simpleboot: Simple Bootstrap Routines*. R package version 1.1-8, <https://github.com/rdpeng/simpleboot>.

### Propensity Score Matching

- Ho D, Imai K, King G, Stuart E (2011). “MatchIt: Nonparametric Preprocessing for Parametric Causal Inference.” *Journal of Statistical Software*, *42*(8), 1-28. doi:10.18637/jss.v042.i08 <https://doi.org/10.18637/jss.v042.i08>.
- Greifer N (2024). *cobalt: Covariate Balance Tables and Plots*. R package version 4.5.5, <https://github.com/ngreifer/cobalt>, <https://ngreifer.github.io/cobalt/>.
